# O-GlcNAcylation of MORC2 at threonine 556 by OGT couples TGF-β signaling to breast cancer progression

**DOI:** 10.1038/s41418-021-00901-0

**Published:** 2022-01-01

**Authors:** Ying-Ying Liu, Hong-Yi Liu, Tian-Jian Yu, Qin Lu, Fang-Lin Zhang, Guang-Yu Liu, Zhi-Ming Shao, Da-Qiang Li

**Affiliations:** 1 Fudan University Shanghai Cancer Center and Shanghai Key Laboratory of Medical Epigenetics, International Co-laboratory of Medical Epigenetics and Metabolism, Ministry of Science and Technology, Institutes of Biomedical Sciences, Fudan University, Shanghai, 200032 China; 2 Cancer Institute, Shanghai Medical College, Fudan University, Shanghai, 200032 China; 3 Department of Oncology, Shanghai Medical College, Fudan University, Shanghai, 200032 China; 4 Department of Breast Surgery, Shanghai Medical College, Fudan University, Shanghai, 200032 China; 5 Shanghai Key Laboratory of Breast Cancer, Shanghai Medical College, Fudan University, Shanghai, 200032 China; 6 Shanghai Key Laboratory of Radiation Oncology, Shanghai Medical College, Fudan University, Shanghai, 200032 China

**Keywords:** Oncogenes, Metastasis

## Abstract

MORC family CW-type zinc finger 2 (MORC2) is a newly identified chromatin-remodeling enzyme involved in DNA damage response and gene transcription, and its dysregulation has been linked with Charcot-Marie-Tooth disease, neurodevelopmental disorder, and cancer. Despite its functional importance, how MORC2 is regulated remains enigmatic. Here, we report that MORC2 is O-GlcNAcylated by O-GlcNAc transferase (OGT) at threonine 556. Mutation of this site or pharmacological inhibition of OGT impairs MORC2-mediated breast cancer cell migration and invasion in vitro and lung colonization in vivo. Moreover, transforming growth factor-β1 (TGF-β1) induces MORC2 O-GlcNAcylation through enhancing the stability of glutamine-fructose-6-phosphate aminotransferase (GFAT), the rate-limiting enzyme for producing the sugar donor for OGT. O-GlcNAcylated MORC2 is required for transcriptional activation of TGF-β1 target genes connective tissue growth factor (CTGF) and snail family transcriptional repressor 1 (SNAIL). In support of these observations, knockdown of GFAT, SNAIL or CTGF compromises TGF-β1-induced, MORC2 O-GlcNAcylation-mediated breast cancer cell migration and invasion. Clinically, high expression of OGT, MORC2, SNAIL, and CTGF in breast tumors is associated with poor patient prognosis. Collectively, these findings uncover a previously unrecognized mechanistic role for MORC2 O-GlcNAcylation in breast cancer progression and provide evidence for targeting MORC2-dependent breast cancer through blocking its O-GlcNAcylation.

## Introduction

Cancer cells exert malignant phenotypes partially through reprogramming the posttranslational modification (PTM) patterns of cancer-relevant proteins in response to extracellular and intracellular stimuli [1]. One of such prevalent PTMs is O-linked N-acetylglucosaminylation (O-GlcNAcylation), which is controlled by the concerted actions of a single pair of opposing enzymes, termed O-linked N-acetylglucosamine (O-GlcNAc) transferase (OGT) and O-GlcNAcase (OGA) [2, 3]. OGT transfers a single O-GlcNAc moiety to the hydroxyl group of serine or threonine residues of substrate proteins from the direct donor uridine diphosphate N-acetylglucosamine (UDP-GlcNAc), whereas OGA catalyzes the opposite reaction to cleave O-GlcNAc from O-GlcNAcylated proteins [2, 3].

In addition to OGT and OGA, the cycling of protein O-GlcNAcylation is dependent on the intracellular concentration of UDP-GlcNAc, the final product of hexosamine biosynthetic pathway (HBP) [4]. Glutamine-fructose-6-phosphate aminotransferase (GFAT) is the rate-limiting enzyme of HBP, which converts fructose-6-phosphate, an intermediate metabolite in the glycolytic pathway and the first substrate for HBP, to glucosamine-6-phosphate (GlcN-6P). Subsequent acetylation and uridylation of GlcN-6P produce UDP-GlcNAc [4]. Emerging evidence shows that transcription factor CCAAT/enhancer-binding protein B (CEBPB) [5], spliced X-box binding protein 1 (Xbp1s) [6], and multiple extracellular and intracellular signals, such as hypoxia, glucose, glucosamine, nicotine, picolinic acid, and epithelial growth factor [5, 7–9], transcriptionally regulate GFAT expression. In addition, phosphorylation of GFAT by AMP-activated protein kinase (AMPK) [10, 11], calcium/calmodulin-dependent kinase II (CAMK-II) [11], and cAMP-dependent protein kinase (PKA) [12] modulates its enzymic activity. Although GFAT has been documented to be a short-lived protein [13], the underlying mechanisms for controlling its protein turnover remain unknown.

O-GlcNAcylation modification as a signaling moiety alters the functions and activities of target proteins and has extensive cross-talks with other PTMs, such as phosphorylation [3, 4]. Consequently, deregulation of O-GlcNAc cycling has been implicated in the pathogenesis of a plethora of chronic diseases including cancer [4]. In this context, increased levels of O-GlcNAcylation, OGT or GFAT in cancer patients are correlated with cancer progression and a poor prognosis [14]. Pharmacological inhibition of OGT or GFAT suppresses cancer progression and enhances cellular sensitivity to anticancer agents [15, 16]. Therefore, identification of novel O-GlcNAc-modified substrates not only will lead to a better understanding of the functions of protein O-GlcNAcylation, but also is helpful for the development of new strategies for treating and preventing cancer [4].

Accumulating evidence indicates that chromatin-remodeling proteins have fundamental roles in cancer progression and therapeutic responsiveness through integrating the extracellular and intracellular signals to control gene transcription and DNA damage response [17]. A case in point is MORC family CW-type zinc finger 2 (MORC2), a member of the highly conserved microrchidia (MORC) ATPase protein family [18]. MORC2 protein contains a catalytically active ATPase module, a CW-type zinc finger domain, a chromo-like domain, and four coiled-coil domains [18–20]. We and others recently demonstrated that MORC2 exerts an ATPase-dependent chromatin remodeling activity and is involved in DNA damage response [21] and gene transcription [19, 22]. The functional importance of MORC2 in human diseases is highlighted by the fact that its mutations have been linked with Charcot-Marie-Tooth disease [23–25], neurodevelopmental disorder [26], and cancer [27]. In addition, MORC2 is upregulated in a variety of human tumors and contributes to their aggressive phenotypes [28, 29]. Recent work from our group shows that MORC2 promotes breast cancer progression and resistance to endocrine therapy and DNA-damaging chemotherapy and radiotherapy [21, 27, 30–32]. Despite its functional importance in human cancer, the regulatory mechanism of MORC2 still remains enigmatic.

In this study, we provide the first evidence that MORC2 is O-GlcNAcylated by OGT at the conserved threonine 556 (T556). Moreover, transforming growth factor-β1 (TGF-β1) induces MORC2 O-GlcNAcylation through enhancing the stability of GFAT. O-GlcNAcylated MORC2 governs the expression of TGF-β1 target genes connective tissue growth factor (CTGF) and snail family transcriptional repressor 1 (SNAIL) and is crucial for breast cancer progression. These findings reveal novel mechanistic insights into MORC2-mediated breast cancer progression and provide potential therapeutic opportunities for MORC2-dependent breast cancer through blocking its O-GlcNAcylation.

## Results

### OGT interacts with MORC2 in breast cancer cells

**Fig. 1:**
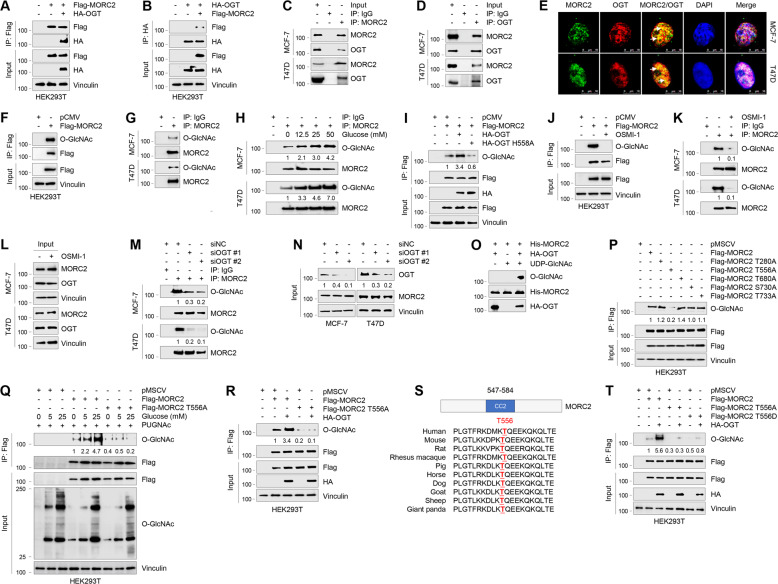
OGT interacts with MORC2 and O-GlcNAcylates MORC2 at T556. **A**, **B** HEK293T cells were transfected with Flag-MORC2 and HA-OGT alone or in combination. IP and immunoblotting analyses were performed with the indicated antibodies after 48 h of transfection. **C**, **D** Lysates from MCF-7 and T47D cells were subjected to IP and immunoblotting analysis with the indicated antibodies. **E** Immunofluorescence staining of MORC2 and OGT in MCF-7 and T47D cells. Cell nucleus was counterstained with DAPI. **F** HEK293T cells were transfected with pCMV or Flag-MORC2. IP and immunoblotting analyses were performed with the indicated antibodies after 48 h of transfection. **G** Lysates from MCF-7 and T47D cells were subjected to IP and immunoblotting analysis with the indicated antibodies. **H** MCF-7 and T47D cells were cultured in glucose- and serum-free medium for 24 h, and treated with increasing doses of glucose for 24 h. IP and immunoblotting analyses were performed with the indicated antibodies. O-GlcNAc levels were normalized to levels of immunoprecipitated MORC2. The input concerning this experiment is shown in Supplementary Fig. S1F. **I** HEK293T cells were transfected with Flag-MORC2, HA-OGT or HA-OGT H558A alone or in combination. After 48 h of transfection, cells were subjected to IP and immunoblotting analysis. O-GlcNAc levels were normalized to levels of immunoprecipitated Flag-MORC2. **J** HEK293T cells transfected with pCMV or Flag-MORC2 were treated with or without 50 μM OSMI-1 for 24 h, and then subjected to IP and immunoblotting analyses with the indicated antibodies. **K**, **L** MCF-7 and T47D cells were treated with or without 50 μM OSMI-1 for 24 h and subjected to IP and immunoblotting analyses with the indicated antibodies. In K, O-GlcNAc levels were normalized to levels of immunoprecipitated MORC2. **M**, **N** MCF-7 and T47D cells were transfected with negative control siRNA (siNC) or two independent siRNAs targeting OGT (siOGT). After 48 h of transfection, cells were subjected to IP and immunoblotting analysis with the indicated antibodies. O-GlcNAc levels were normalized to levels of immunoprecipitated MORC2 (M), and OGT levels were normalized to those of Vinculin (N). **O** HA-OGT was purified from HEK293T transfected with HA-OGT. Purified His-MORC2 were incubated with HA-OGT in reaction buffer in a final volume of 25 μl per sample. The samples were incubated at 37 °C for 24 h. MORC2 O-GlcNAc was detected by immunoblotting with an anti-O-GlcNAc antibody (RL2). **P** HEK293T cells were transfected with the indicated expression vectors. After 48 h of transfection, cells were subjected to IP and immunoblotting analysis with the indicated antibodies. O-GlcNAc levels were normalized to levels of immunoprecipitated Flag-MORC2. **Q** HEK293T cells were transfected with the indicated expression vectors. After 24 h of transfection, cells were cultured in glucose- and serum-free medium for 24 h and then treated with increasing doses of glucose for 24 h. IP and immunoblotting analyses were performed with the indicated antibodies. O-GlcNAc levels were normalized to levels of immunoprecipitated Flag-MORC2. **R** HEK293T cells were transfected with Flag-MORC2 or Flag-MORC2 T556A alone or in combination with HA-OGT. After 48 h of transfection, cells were subjected to IP and immunoblotting analysis. O-GlcNAc levels were normalized to levels of immunoprecipitated Flag-MORC2. **S** Alignment of OGT protein sequence among different species. **T** HEK293T cells were transfected with Flag-MORC2 (WT, T556A, or T556D) alone or in combination with HA-OGT. After 48 h of transfection, cells were subjected to IP and immunoblotting analysis. O-GlcNAc levels were normalized to levels of immunoprecipitated Flag-MORC2.

To gain mechanistic insights into the biological functions of MORC2, we recently performed immunoprecipitation (IP) coupled with lipid chromatography-tandem mass spectrometry to characterize MORC2 interacting proteins [32], and identified OGT as a potential binding partner of MORC2 (Supplementary Fig. S1A). To validate this result, HEK293T cells were transfected with Flag-MORC2, HA-OGT alone or in combination, and then subjected to reciprocal IP assays with an anti-Flag or anti-HA antibody. Immunoblotting analysis with the indicated antibodies revealed that Flag-MORC2 was co-immunoprecipitated with HA-OGT when both were co-expressed (Fig. 1A, B). Moreover, there was an interaction between MORC2 and OGT at the endogenous protein level in MCF-7 and T47D cells (Fig. 1C, D). Immunofluorescent staining showed a partial co-localization between MORC2 and OGT in both cell lines (Fig. 1E). Domain-mapping experiments using various deletion constructs further demonstrated that the interaction between MORC2 and OGT was mediated by the N-terminal region of MORC2 (residues 1–420) (Supplementary Fig. S1B, S1C) and the C-terminal domain of OGT (residues 901–1046) (Supplementary Fig. S1D, S1E). These results suggest that OGT interacts with MORC2.

### OGT O-GlcNAcylates MORC2 at the conserved threonine 556

As OGT is the unique enzyme for catalyzing O-GlcNAcylation of intracellular proteins [3], we next examined whether MORC2 is modified by O-GlcNAcylation. Toward this aim, HEK293T cells were transfected with Flag-MORC2 and then subjected to IP assays with an anti-Flag antibody. Immunoblotting with a mouse monoclonal anti-O-GlcNAc antibody (RL2) revealed a positive O-GlcNAc signal in the immunoprecipitated MORC2 (Fig. 1F). Moreover, IP assays using an anti-MORC2 antibody revealed that endogenous MORC2 was O-GlcNAcylated in MCF-7 and T47D cells (Fig. 1G). Treatment of MCF-7 and T47D cells with glucose, which produces the donor substrate UDP-GlcNAc for OGT through HBP [3], resulted in an increase in MORC2 O-GlcNAcylation in a dose-dependent manner (Fig. 1H and Supplementary Fig. S1F). These results suggest that MORC2 is an O-GlcNAcylated protein.

We next investigated the involvement of OGT in the noted MORC2 O-GlcNAcylation. As shown in Fig. 1I, ectopic expression of wild-type (WT) OGT, but not its catalytically inactive mutant (H558A) [33], enhanced O-GlcNAcylation of exogenously expressed MORC2 in HEK293T cells. Consistently, inhibition of OGT activity by small molecule inhibitor OSMI-1 [34] reduced O-GlcNAcylation levels of exogenous MORC2 in HEK293T cells (Fig. 1J) and of endogenous MORC2 in MCF-7 and T47D cells (Fig. 1K, L). These results were further demonstrated by knockdown of endogenous OGT in MCF-7 and T47D cells using two independent siRNAs (Fig. 1M, N). In vitro O-GlcNAcylation assays using bacterially expressed His-MORC2 and purified HA-OGT protein from HEK293T cells demonstrated that OGT O-GlcNAcylated MORC2 in the presence of UDP-GlcNAc (Fig. 1O).

To identify the site(s) of MORC2 O-GlcNAcylation, we transiently co-expressed Flag-MORC2 and HA-OGT in HEK293T cells. After immunoprecipitation using anti-Flag M2 beads and in-gel trypsin digestion, resultant peptides were subjected to electron transfer dissociation-mass spectrometry analysis [35]. By this approach, we identified five potential O-GlcNAcylation sites on MORC2, including T280, T556, T680, S730, and T733 (Supplementary Fig. S2). To determine which residue is the major O-GlcNAcylation site in MORC2, we individually mutated those five residues to alanine (A), which lacks the hydroxy group in the side chain and thus blocks O-GlcNAc attachment [36], and then transfected them into HEK293T cells. Sequential IP and immunoblotting analyses with the indicated antibodies showed that mutation of T556, but not the other four residues, resulted in a decrease in MORC2 O-GlcNAcylation levels (Fig. 1P). Moreover, treatment with glucose (Fig. 1Q) or ectopic expression of OGT (Fig. 1R) enhanced the O-GlcNAcylation levels of WT MORC2 in comparison with T556A mutant. These results suggest that T556 is the primary O-GlcNAcylation site in MORC2. Sequence alignment revealed that the T556 residue is located within the second coiled-coil domain (residues 547–584) of MORC2 and is highly conserved across species (Fig. 1S). To determine whether potential T556 phosphorylation of MORC2 affects its O-GlcNAcylation, we replaced T556 residue with aspartic acid (T556D) to mimic a constitutively phosphorylated MORC2 at T556. As shown in Fig. 1T, expression of phospho-mimetic T556D mutant or phospho-deficient T556A mutant significantly decreased the levels of MORC2 O-GlcNAcylation. Collectively, these results indicate that T556 is the main O-GlcNAcylation site in MORC2.

### OGA de-O-GlcNAcylates MORC2 at T556

**Fig. 2:**
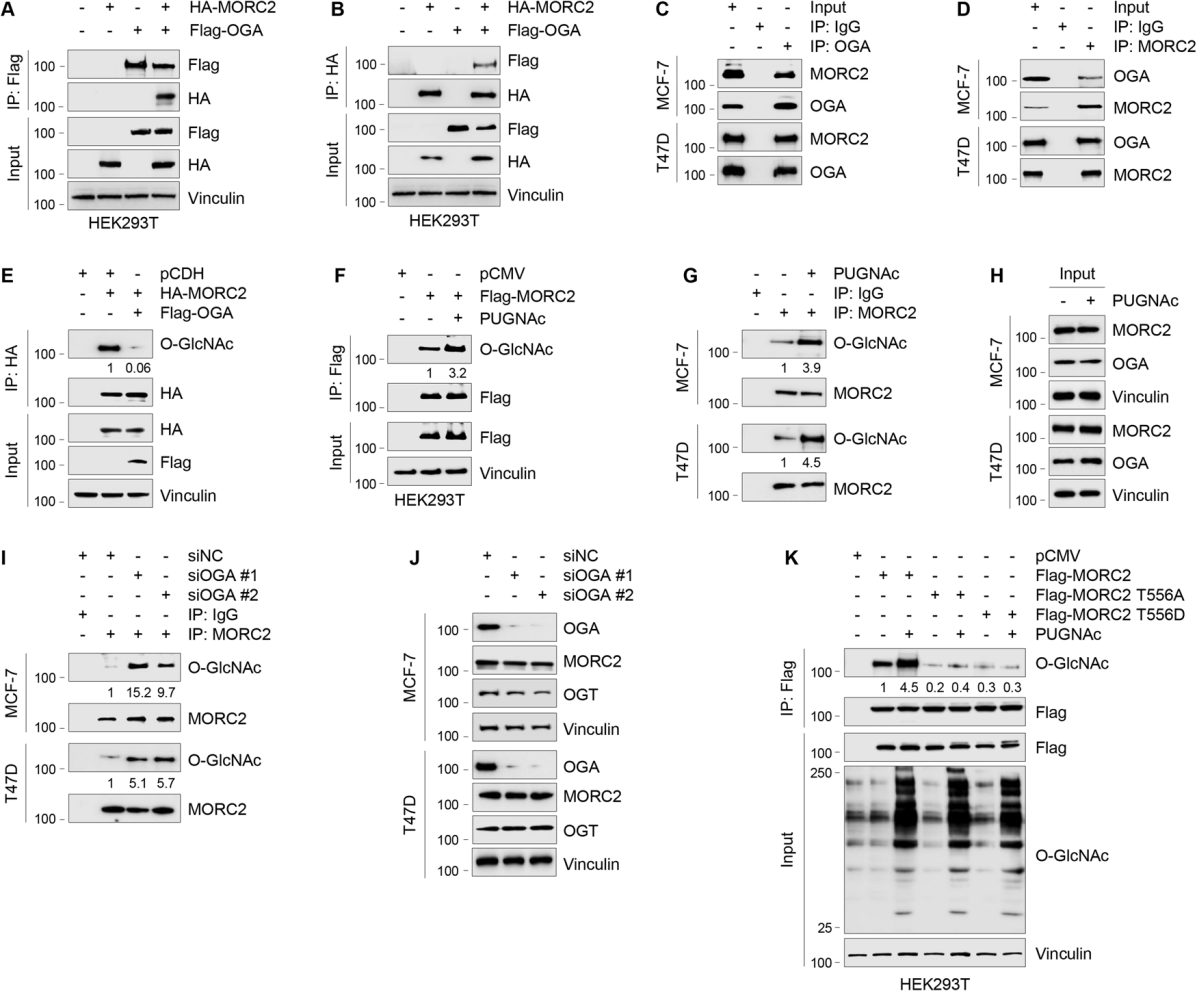
OGA interacts with MORC2 and de-O-GlcNAcylates MORC2 at T556. **A**, **B** HEK293T cells were transfected with HA-MORC2 and Flag-OGA alone or in combination. IP and immunoblotting analysis were performed with the indicated antibodies after 48 h of transfection. **C**, **D** Lysates from MCF-7 and T47D cells were subjected to IP and immunoblotting analysis with the indicated antibodies. **E** HEK293T cells were transfected with Flag-OGA and HA-MORC2 alone or in combination. IP and immunoblotting analysis were performed with the indicated antibodies after 48 h of transfection. O-GlcNAc levels were normalized to levels of immunoprecipitated HA-MORC2. **F** HEK293T cells transfected with pCMV or Flag-MORC2 were treated with or without 20 μM PUGNAc for 24 h and subjected to IP and immunoblotting analyses with the indicated antibodies. O-GlcNAc levels were normalized to levels of immunoprecipitated Flag-MORC2. **G**, **H** MCF-7 and T47D cells were treated with or without 20 μM PUGNAc for 24 h and subjected to IP and immunoblotting analyses with the indicated antibodies. O-GlcNAc levels were normalized to levels of immunoprecipitated MORC2. **I**, **J** MCF-7 and T47D cells were transfected with siNC or two different siRNAs targeting OGA (siOGA). After 48 h of transfection, cells were subjected to IP and immunoblotting analysis with the indicated antibodies. In I, O-GlcNAc levels were normalized to levels of immunoprecipitated MORC2. **K** HEK293T cells were transfected with the indicated expression vectors. After 24 h of transfection, cells were treated with or without 20 μM PUGNAc for 24 h and subjected to IP and immunoblotting analysis. O-GlcNAc levels were normalized to levels of immunoprecipitated Flag-MORC2.

O-GlcNAcylation is a dynamic and reversible process, and the glycosylase catalyzing protein de-O-GlcNAcylation is OGA [2, 3]. As shown in Fig. 2A–D, we demonstrated that MORC2 interacted with OGA at both exogenous and endogenous levels. Moreover, ectopic expression of OGA decreased MORC2 O-GlcNAcylation (Fig. 2E). Conversely, inhibition of OGA activity by small molecule inhibitor PUGNAc [37] enhanced the O-GlcNAcylation levels of exogenously expressed MORC2 in HEK293T cells (Fig. 2F) and of endogenous MORC2 in MCF-7 and T47D cells (Fig. 2G, H). Consistently, knockdown of OGA in MCF-7 and T47D cells using two independent siRNAs reduced the O-GlcNAcylation levels of endogenous MORC2 (Fig. 2I, J). Moreover, treatment with OGA inhibitor PUGNAc enhanced the O-GlcNAcylation levels of WT MORC2, but not its T556A or T556D mutant (Fig. 2K). Together, these results suggest that OGA de-O-GlcNAcylates MORC2 at T556.

### OGT inhibitor OSMI-1 blocks MORC2-mediated breast cancer progression

**Fig. 3:**
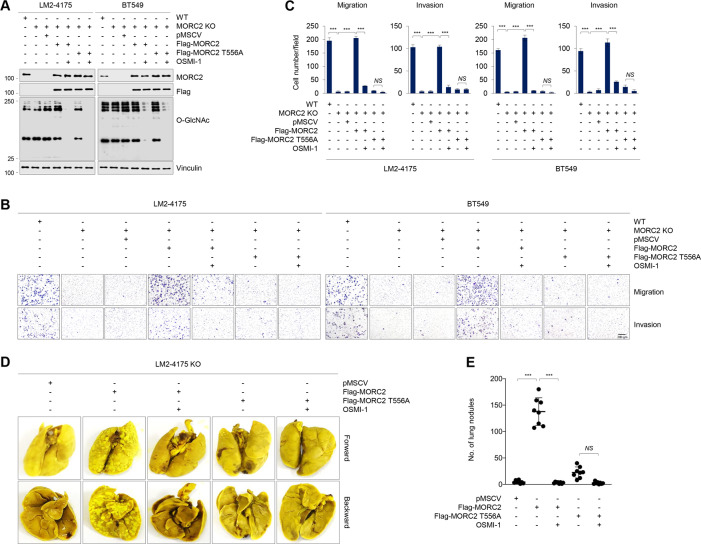
O-GlcNAcylated MORC2 contributes to breast cancer progression. **A**–**C** WT and MORC2-KO LM2–4175 and BT549 cells stably expressing pMSCV, Flag-MORC2 or Flag-MORC2 T556A were treated with or without 50 μM OSMI-1 for 24 h, and then subjected to immunoblotting analysis with the indicated antibodies (A) or Transwell migration and invasion assays as described in Materials and Methods. Representative images of migrated and invaded cells (B) and corresponding quantitative results (**C**) are shown. ***, *p* < 0.001; *NS*, no significance. **D**, **E** WT and MORC2-KO LM2–4175 and BT549 cells stably expressing pMSCV, Flag-MORC2 or Flag-MORC2 T556A were injected into 6-week-old female BALB/c nude mice (*n* = 8) through the tail vein. OSMI-1 treatment was started 1 week after injection. OSMI-1 was given at a dose of 10 mg/kg/day for four weeks, five days a week and two days off at weekends. After 5 weeks of injections, the lungs were harvested. Representative images of lung metastasis (D), and quantitative results of lung nodules (E) are shown. ***, *p* < 0.001; *NS*, no significance.

To address the impact of MORC2 O-GlcNAcylation on biological behavior of breast cancer cells, we first knocked out (KO) endogenous MORC2 in LM2–4175 and BT549 cells using CRISPR-Cas9 technology [38], and then reintroduced empty vector pMSCV or Flag-MORC2 (WT, T556A or T556D) into resultant MORC2 KO cells by lentiviral infection (Fig. 3A). Transwell migration and Matrigel invasion assays showed that knockout of MORC2 reduced the migratory and invasive potential of LM2–4175 and BT549 cells, which was partially restored after re-expression of WT MORC2, but not either T556A (Fig. 3B, C) or T556D (Supplementary Fig. S3) mutant, in MORC2-KO LM2–4175 and BT549 cells. Moreover, treatment with OGT inhibitor OSMI-1 [34] suppressed WT MORC2-mediated breast cancer cell migration and invasion (Fig. 3B, C). In vivo mouse xenograft studies demonstrated that mice injected with cells expressing T556A mutant MORC2 had less metastatic nodules compared to those injected with WT MORC2 expressing cells (Fig. 3D, E). Moreover, administration of OGT inhibitor OSMI-1 suppressed lung metastasis in mice injected with cells expressing WT MORC2 (Fig. 3D, E). Collectively, these results suggest that MORC2 O-GlcNAcylation is critical for breast cancer progression, which can be blocked by OGT inhibitor OSMI-1.

### TGF-β1 induces MORC2 O-GlcNAcylation at T556 through enhancing GFAT stability

**Fig. 4:**
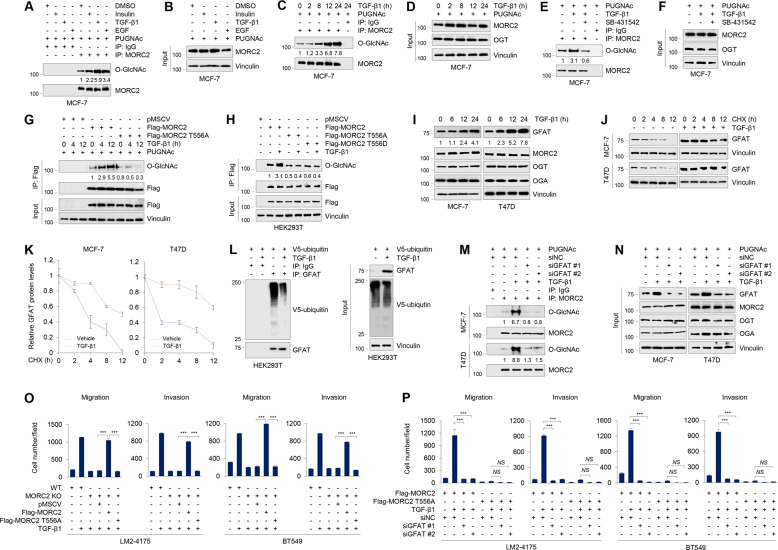
TGF-β1 induces MORC2 O-GlcNAcylation at T556 through enhancing GFAT stability. **A**, **B** MCF-7 cells were serum-starved for 24 h and then treated with or without 10 μg/ml insulin, 5 ng/ml TGF-β1, or 20 ng/ml EGF for another 24 h. Cells were harvested for IP and immunoblotting analysis with the indicated antibodies. In A, O-GlcNAc levels were normalized to levels of immunoprecipitated MORC2 levels. **C**, **D** MCF-7 cells were serum-starved for 24 h, and then treated with or without 5 ng/ml TGF-β1 for the indicated times. Cell lysates were subjected to IP and immunoblotting analysis with the indicated antibodies. In C, O-GlcNAc levels were normalized to levels of immunoprecipitated MORC2. **E**, **F** MCF-7 cells were serum-starved for 24 h, and then treated with 5 ng/ml TGF-β1 (24 h) alone or in combination with 5 μM TGF-β inhibitor SB431542 (12 h). Cells were harvested for IP and immunoblotting analysis with the indicated antibodies. In E, O-GlcNAc levels were normalized to levels of immunoprecipitated MORC2. **G**, **H** HEK293T cells were transfected with the indicated expression vectors. After 24 h of transfection, cells were serum-starved for 24 h, followed by treatment with 5 ng/ml TGF-β1 for the indicated times. IP and immunoblotting analysis were performed with the indicated antibodies. In G, O-GlcNAc levels were normalized to levels of immunoprecipitated Flag-MORC2. **I** MCF-7 and T47D cells were serum-starved for 24 h and then treated with or without 5 ng/ml TGF-β1 for the indicated times. Immunoblotting analyses were performed with the indicated antibodies. GFAT levels were normalized to Vinculin levels. **J, K** MCF-7 and T47D cells were serum-starved for 24 h and then treated with or without 5 ng/ml TGF-β1 for 24 h. Cells were treated with 100 μg/ml of cycloheximide (CHX) for the indicated times and then analyzed by immunoblotting (J). Relative expression levels of GFAT to Vinculin are shown in (K). **L** HEK293T cells were transfected with V5-ubiquitin. After 24 h of transfection, cells were serum-starved for 24 h and then treated with or without 5 ng/ml TGF-β1 for another 24 h. IP and immunoblotting analyses were performed with the indicated antibodies. **M**, **N** MCF-7 and T47D cells were transfected with siNC or two different siRNAs targeting GFAT (siGFAT). After 24 h of transfection, cells were serum-starved for 24 h and then treated with or without 5 ng/ml TGF-β1 for another 24 h. IP and immunoblotting analyses were performed with the indicated antibodies. In M, O-GlcNAc levels were normalized to levels of immunoprecipitated MORC2. **O** WT and MORC2-KO LM2–4175 and BT549 cells stably expressing pMSCV, Flag-MORC2 or Flag-MORC2 T556A were serum-starved for 24 h, treated with or without 5 ng/ml TGF-β1 for 24 h, and then subjected to Transwell migration and invasion assays. Corresponding quantitative results are shown in O. ***, *p* < 0.001. Representative images of migrated and invaded cells are shown in Supplementary Fig. S7D. **P** MORC2-KO LM2–4175 and BT549 cells stably expressing Flag-MORC2 or Flag-MORC2 T556A were transfected with siNC or two siRNAs targeting GFAT (siGFAT). After 24 h of transfection, cells were serum-starved for 24 h, followed by treatment with or without 5 ng/ml TGF-β1 for 24 h. Transwell migration and invasion assays were performed as described in Materials and Methods. Corresponding quantitative results are shown in P. **p < 0.01. ***, *p* < 0.001; *NS*, no significance. Representative images of migrated and invaded cells are  shown in Supplementary Fig. S7F.

Protein O-GlcNAcylation is inducible in response to various extracellular or intracellular signals [3]. To investigate the upstream regulatory signals for MORC2 O-GlcNAcylation, we treated MCF-7 cells with three ubiquitous growth factors, including insulin, TGF-β1, and epidermal growth factor (EGF), and then determined their impacts on endogenous MORC2 O-GlcNAcylation. Results showed that all of them enabled to induce MORC2 O-GlcNAcylation, and TGF-β1 showed the strongest response, followed by EGF and insulin (Fig. 4A, B). Furthermore, MORC2 O-GlcNAcylation was enhanced in MCF-7 cells following TGF-β1 treatment in a time-dependent manner (Fig. 4C, D). Induction of MORC2 O-GlcNAcylation by TGF-β1 was also observed in T47D cells (Supplementary Fig. S4A, S4B). In agreement with these observations, preincubation of TGF-β inhibitor SB431542 [39] attenuated TGF-β1-induced MORC2 O-GlcNAcylation in MCF-7 (Fig. 4E, F) and T47D (Supplementary Fig. S4C, S4D) cells. Moreover, the O-GlcNAcylation levels of WT MORC2, but not its T556A or T556D mutant, were increased after treatment with TGF-β1 (Fig. 4G, H). These results suggest that TGF-β1 is one of the upstream regulatory signals for MORC2 T556 O-GlcNAcylation.

To investigate the molecular mechanism by which TGF-β1 induces MORC2 O-GlcNAcylation, we first examined whether TGF-β1 affects the interaction of MORC2 with OGT or OGA by IP assays. As shown in Supplementary Fig. S4E–S4G, treatment of MCF-7 and T47D cells with TGF-β1 did not significantly affect the binding of MORC2 with OGT or OGA. Interestingly, it was recently documented that treatment of human lung cancer A549 cells with TGF-β1 results in an increase in GFAT expression levels [40], but how this occurs remains unexplored. GFAT is the rate-limiting enzyme in HBP for producing the direct donor UDP-GlcNAc for OGT-mediated protein O-GlcNAcylation [3, 4]. Immunoblotting analyses showed that treatment with TGF-β1 resulted in an increase in protein levels of GFAT, but not MORC2, OGT or OGA (Fig. 4I). qPCR analysis showed that the mRNA levels of TGF-β1 target gene SNAIL, but not GFAT or MORC2, were upregulated following TGF-β1 treatment (Supplementary Fig. S5A). These results indicate the effects of TGF-β1 on GFAT upregulation to be post-transcriptional.

As GFAT is a short-lived protein [13], we next explored the potential role of the proteasome pathway in controlling its steady-state levels. As shown in Supplementary Fig. S5B, protein levels of GFAT were increased in HEK293T cells after treatment with proteasome inhibitor MG-132. As a control, treatment with MG-132 did not significantly affect the protein levels of MORC2, which is known to be degraded through the autophagy-lysosome but not ubiquitin-proteasome pathway [31]. Cycloheximide (CHX) chase assays revealed that TGF-β1 treatment enhanced the half-life of GFAT protein (Fig. 4J, K). IP and immunoblotting analysis with the indicated antibodies showed a significant decrease in polyubiquitination levels of GFAT in TGF-β1-treated cells (Fig. 4L). These results suggest that TGF-β1 stabilizes GFAT by blocking its proteasomal degradation. Furthermore, knockdown of GFAT using two independent siRNAs impaired TGF-β1-induced MORC2 O-GlcNAcylation in MCF-7 and T47D cells (Fig. 4M, N).

TGF-β1 is frequently overexpressed in human breast tumors and tumor-associated stroma and its upregulation closely correlates with breast cancer cell motility, invasion, and metastasis [41–43]. To address whether MORC2 O-GlcNAcylation is involved in TGF-β1-mediated breast cancer cell migration and invasion, we reconstituted empty vector pMSCV or Flag-MORC2 (WT or T556A) into MORC2-KO LM2–4175 and BT549 cells by lentiviral infection and then treated with or without TGF-β1 (Supplementary Fig. S5C). Transwell migration and invasion assays showed that treatment with TGF-β1 significantly enhanced the migratory and invasive potential of WT LM2–4175 and BT549 cells relative to MORC2 KO counterparts (Fig. 4O and Supplementary Fig. S5D). Moreover, reconstitution of WT, but not T556A mutant, MORC2, into MORC2-KO LM2–4175 and BT549 cells partially restored the responsiveness of MORC2 KO cells to TGF-β1 treatment (Fig. 4O and Supplementary Fig. S5D). Moreover, knockdown of GFAT using two independent siRNAs (Supplementary Fig. S5E) impaired TGF-β1-induced enhancement of migrative and invasive capacity of MORC2-KO LM2–4175 and BT549 cells re-expressing WT MORC2 (Fig. 4P and Supplementary Fig. S5F). These results suggest that TGF-β1 induces O-GlcNAcylation of MORC2 through enhancing the stability of GFAT.

### O-GlcNAcylated MORC2 regulates CTGF and SNAIL expression

**Fig. 5:**
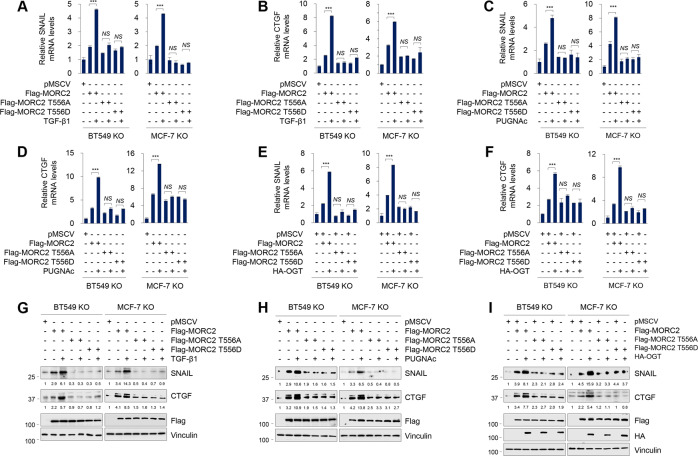
O-GlcNAcylated MORC2 transcriptionally regulates CTGF and SNAIL expression. **A**, **B** MORC2-KO MCF-7 and BT549 cells stably expressing pMSCV or Flag-MORC2 (WT, T556A, or T556D) were serum-starved for 24 h, treated with or without 5 ng/ml TGF-β1 for another 24 h, and then subjected to qPCR analysis of SNAIL (A) or CTGF (B) mRNA levels. ***, *p* < 0.001; *NS*, no significance. **C**, **D** MORC2-KO MCF-7 and BT549 cells stably expressing pMSCV or Flag-MORC2 (WT, T556A, or T556D) were treated with or without 20 μM PUGNAc for 24 h and then subjected to qPCR analysis of SNAIL (C) or CTGF (D) mRNA levels. ***, *p* < 0.001; *NS*, no significance. **E**, **F** MORC2-KO MCF-7 and BT549 cells stably expressing pMSCV or Flag-MORC2 (WT, T556A, or T556D) were transfected with or without HA-OGT. After 48 h of transfection, cells were subjected to qPCR analysis of SNAIL (E) or CTGF (F) mRNA levels. ***, *p* < 0.001. *NS*, no significance. **G**, **H** MORC2-KO MCF-7 and BT549 cells stably expressing pMSCV or Flag-MORC2 (WT, T556A or T556D) were treated with or without 5 ng/ml TGF-β1 for 24 h (G) or 20 μM PUGNAc for 24 h (H), then and subjected to immunoblotting analysis. CTGF and SNAIL levels were normalized to Vinculin levels. In G, cells were serum-starved for 24 h prior to TGF-β1 treatment. **I** MORC2-KO MCF-7 and BT549 cells stably expressing pMSCV or Flag-MORC2 (WT, T556A or T556D) were transfected with or without HA-OGT. After 48 h of transfection, cells were subjected to immunoblotting analysis. CTGF and SNAIL levels were normalized to Vinculin levels.

As MORC2 and TGF-β exert their biological functions through, at least in part, regulating gene transcription [19, 22, 44], we first analyzed two publicly available RNA-sequencing (RNA-Seq) datasets, including GSE74377 [45] and GSE95452 [19]. The former includes the mRNA profiles of MCF10A cells with or without TGF-β1 treatment [45], while the latter contains the expression profiling of WT and MORC2-KO HeLa cells [19]. According to the preset threshold, we found 9 commonly upregulated genes by TGF-β1 and MORC2, including SNAIL, CTGF, SPHK1, RHOB, CRYAB, FBN1, PPP2R2B, KIAA1644, and ATP8B2 (Supplementary Fig. S6A). Based on the documented functions of these genes in human cancer (Supplementary Fig. S6B), we selected SNAIL and CTGF for further validation by qPCR assays in MORC2-KO BT549 and MCF-7 cells reconstituted with empty vector pMSCV, WT or T556A mutant MORC2. Results showed that treatment with TGF-β1 (Fig. 5A, B), OGA inhibitor PUGNAc (Fig. 5C, D) or ectopic expression of HA-OGT (Fig. 5E, F) resulted in an upregulation in CTGF and SNAIL mRNA levels in MORC2-KO BT549 and MCF-7 cells re-expressing WT MORC2 relative to those expressing T556A or T556D mutant MORC2. These results were further demonstrated by immunoblotting assays (Fig. 5G–I). The results indicate that MORC2 T556 O-GlcNAcylation transcriptionally activates CTGF and SNAIL expression.

**Fig. 6:**
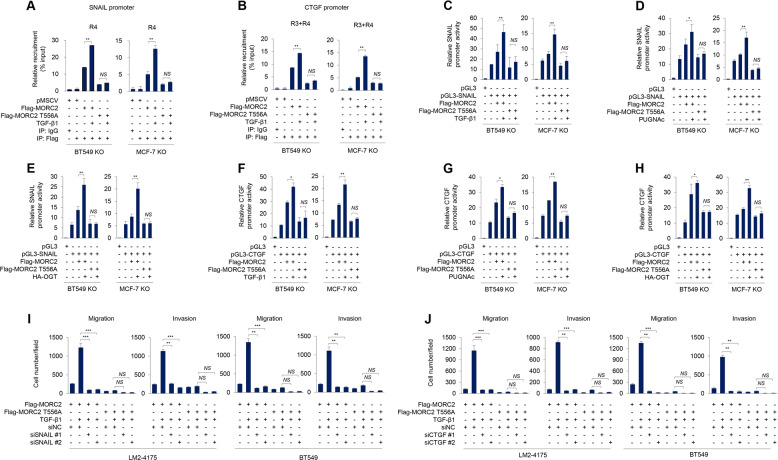
O-GlcNAcylated MORC2 is recruited to SNAIL and CTGF promoters and enhances promoter activities of SNAIL and CTGF. **A**, **B** MORC2-KO BT549 and MCF-7 cells stably expressing pMSCV or Flag-MORC2 (WT or T556A) were serum-starved for 24 h, and then treated with or without 5 ng/ml TGF-β1 for 24 h. ChIP assays were performed with an anti-Flag antibody or IgG, followed by qPCR analysis. Recruitment of Flag-MORC2 to the *SNAIL* (A) or *CTGF* (B) promoter was normalized to Input. **p < 0.01; *NS*, no significance. **C**, **D**, **F**, **G** MORC2-KO MCF-7 and BT549 cells stably expressing pMSCV or Flag-MORC2 (WT or T556A) were transfected with a luciferase reporter construct encoding pGL3, pGL3-CTGF (−901 to −2000) or pGL3-SNAIL ( + 100 to −500). After 24 h of transfection, cells were treated with or without 5 ng/ml TGF-β1 for 24 h (C and F) or 20 μM PUGNAc for 24 h (D and G). In D and G, cells were serum-starved for 24 h prior to TGF-β1 treatment. Luciferase assays were performed as described in Materials and Methods. Transfection efficiency was normalized to co-transfected Renilla (Ren) luciferase. Results represent three independent experiments. Error bars represent SEM. **p < 0.01; *NS*, no significance. **E**, **H** MORC2-KO MCF-7 and BT549 cells stably expressing pMSCV or Flag-MORC2 (WT or T556A) were co-transfected with or without a luciferase reporter construct encoding pGL3, pGL3-SNAIL ( + 100 to −500) (E) or pGL3-CTGF (−901 to −2000) (H) and HA-OGT. After 48 h of transfection, luciferase assays were performed as described above. The results are representative of three independent transfection experiments. Error bars represent SEM. **p < 0.01; *NS*, no significance. **I**, **J** MORC2-KO LM2–4175 and BT549 cells stably expressing Flag-MORC2 or Flag-MORC2 T556A were transfected with siNC or two siRNAs targeting SNAIL (siSNAIL) (Supplementary Fig. S10A) or CTGF (siCTGF) (Supplementary Fig. S10B). After 24 h of transfection, cells were serum-starved for 24 h, followed by treatment with or without 5 ng/ml TGF-β1 for 24 h. Transwell migration and invasion assays were performed as described in Materials and Methods. Corresponding quantitative results are shown in I-J. **p < 0.01. ***, *p* < 0.001; *NS*, no significance. Representative images of migrated and invaded cells are shown in Supplementary Fig. S10C and Fig. S10D.

As SNAIL and CTGF positively regulate each other [46–49], we then focused on addressing the underlying mechanism by which MORC2 T556 O-GlcNAcylation regulates SNAIL and CTGF expression. To examine the recruitment of MORC2 onto SNAIL and CTGF promoter, we treated MORC2-KO BT549 and MCF-7 cells re-expressing empty vector pMSCV and Flag-MORC2 with or without TGF-β1, and then immunoprecipitated crosslinked chromatin with an anti-Flag antibody or IgG as a control. qPCR assays were then performed using primers designed against about every 500 bp region of SNAIL and CTGF promoter (Supplementary Fig. S7A, S7B). Results showed that MORC2 was recruited to one region (R4) of SNAIL promoter (−151 to −476) and two regions (R3 and R4) of CTGF promoter (−1035 to −1235 and −1534 to −1925), and these events were enhanced following TGF-β1 treatment (Fig. 6A and Supplementary Fig. S7C). Furthermore, mutation of O-GlcNAcylation site in MORC2 (T556A) reduced its recruitment to SNAIL promoter (R4) and CTGF promoter (R3 and R4), and this phenomenon was not significantly affected by TGF-β1 treatment (Fig. 6B and Supplementary Fig. S7D). To examine whether CTGF and SNAIL promoter activities are affected by MORC2 O-GlcNAcylation, we generated two luciferase reporter constructs containing R4 of SNAIL promoter (pGL3-SNAIL + 100 to −500) and R3 and R4 of CTGF promoter (pGL3-CTGF −901 to −2000), and then transfected them into MORC2-KO BT549 and MCF-7 cells reconstituted with WT or T556A mutant MORC2. Luciferase reporter assays revealed that treatment with TGF-β1 (Fig. 6C, F), PUGNAc (Fig. 6D, G), or ectopic expression of OGT (Fig. 6E, H) enhanced the promoter activities of SNAIL and CTGF in MORC2-KO BT549 and MCF-7 cells re-expressing WT MORC2 relative to its T556A mutant. These results suggest that MORC2 O-GlcNAcylation could affect SNAIL and CTGF promoter activities. Moreover, knockdown of endogenous SNAIL or CTGF using two independent siRNAs (Supplementary Fig. S8A, S8B) impaired TGF-β1-induced enhancement of migrative and invasive capacity of MORC2-KO LM2–4175 and BT549 cells re-expressing WT MORC2 (Fig. 6I, J and Supplementary Fig. S8C, S8D). Together, these results suggest that GFAT-MORC2 O-GlcNAcylation-SNAIL/CTGF pathway is involved in TGF-β1-induced breast cancer cell migration and invasion.

### High expression of OGT, MORC2, SNAIL, and CTGF in breast tumors is correlated with poor patient prognosis

**Fig. 7:**
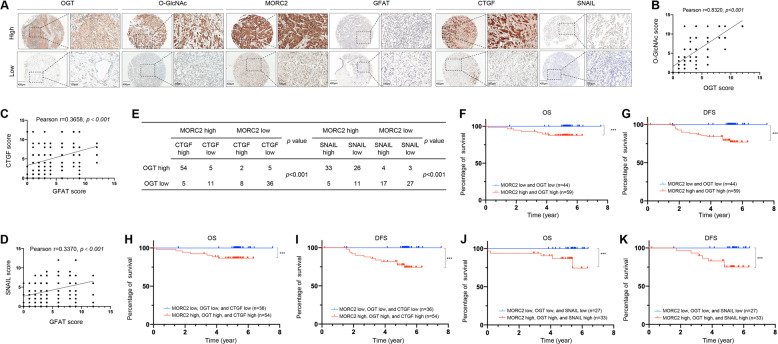
High expression of MORC2, OGT, and SNAIL/CTGF in breast tumors is associated with poor patient prognosis. **A** IHC staining was carried out on a tissue microarray containing 126 breast tumor samples with clinical fellow-up information. Representative IHC images of MORC2, OGT, GFAT, SNAIL, CTGF, and O-GlcNAc expression are shown. Scale bars, 400 μm (left without magnification) and 100 μm (right with magnification). **B**–**D** Correction analysis of OGT and O-GlcNAc (**B**), GFAT and CTGF (**C**), GFAT, and SNAIL (**D**) expression levels in 126 breast tumor tissues. Pearson correlation test was used. **E** Correction analysis of MORC2/OGT/CTGF and MORC2/OGT/SNAIL expression levels in 126 breast tumor tissues. Pearson correlation test was used. **F**, **G** Kaplan–Meier curves of OS (**F**) and DFS (**G**) of 126 breast cancer patients with high or low expression of both MORC2 and OGT proteins are shown. ***, *p* < 0.001. **H**, **I** Kaplan-Meier curves of OS (H) and DFS (I) of 126 breast cancer patients with high or low expression of all of three proteins (MORC2, OGT, and CTGF). **J**, **K** Kaplan–Meier curves of OS (J) and DFS (K) of 126 breast cancer patients with high or low expression of all three proteins (MORC2, OGT, and SNAIL) are shown. ***, *p* < 0.001.

To determine the clinical relevance of our findings, we performed immunohistochemical (IHC) staining using a tissue microarray (TMA) containing 126 human breast tumor samples with an antibody against MORC2, O-GlcNAc, OGT, GFAT, SNAIL, or CTGF. Characterization of clincopathological features of these patients is shown in Supplementary Table S1. Representative IHC images of staining intensity of those six proteins are shown in Fig. 7A. Quantitative results showed that there was a positive correlation in expression levels between OGT and O-GlcNAcylation (Fig. 7B), GFAT and CTGF (Fig. 7C), GFAT and SNAIL (Fig. 7D), MORC2, OGT, and CTGF, or MORC2, OGT and SNAIL (Fig. 7E) in these samples. Due to the well-recognized technical difficulty in generating O-GlcNAc site-specific antibodies at present [50], we utilized the co-expression status of MORC2 and OGT to indirectly reflect the levels of MORC2 T556 O-GlcNAcylation. The Kaplan-Meier method and log-rank test showed that patients whose tumors had high expression of both MORC2 and OGT proteins had poorer overall survival (OS) and disease-free survival (DFS) than those with low expression of MORC2 and OGT (Fig. 7F, G). Similarly, OS and DFS of the patients with high expression of all of three proteins (MORC2, OGT, and CTGF or MORC2, OGT, and SNAIL) were shorter than those of patients with low proteins expression of all of three proteins (Fig. 7H–K). Taken together, these results support the functional role of OGT-MORC2 O-GlcNAcylation-CTGF/SNAIL axis in clinical breast cancer progression.

## Discussion

In this study, we uncovered several interesting findings concerning the functional and mechanistic role of OGT-mediated MORC2 O-GlcNAcylation in breast cancer progression (Supplementary Fig. S9).

First, O-GlcNAcylation is a novel PTM of MORC2, which is required for breast cancer progression. MORC2 is an emerging oncoprotein that is upregulated in various types of human cancer and contributes to cancer cell growth, metastasis, and chemoresistance [27–29, 51]. We recently demonstrated that MORC2 undergoes signaling-dependent phosphorylation [31], acetylation [30], and poly(ADP-ribosyl)ation [32], and these PTMs are implicated in the resistance of breast cancer cells to endocrine therapy drugs and DNA-damaging therapeutic agents. In this study, we provide the first evidence that MORC2 is subjected to O-GlcNAcylation modification at T556, and this process is regulated by OGT and OGA (Figs. 1 and 2). These results add another layer of complexity to the PTM code in regulating biological functions of MORC2. Although many proteins are known to undergo O-GlcNAcylation, only a few site-specific functions for protein O-GlcNAcylation have been reported, including Tau (S400) [52], SIRT1 (S549) [53], TAB1 (S395) [54], c-Myc (T58) [55], and IRS-1 (S1011) [56]. In this study, we showed that mutation of MORC2 O-GlcNAcylation site (T556A) or pharmacological inhibition of OGT by OSMI-1 impairs MORC2-mediated breast cancer cell migration and invasion in vitro and lung colonization in a mouse xenograft mouse model (Fig. 3). These results suggest that MORC2 O-GlcNAcylation contributes to breast cancer progression, which is blocked by OGT inhibitor OSMI-1.

Second, TGF-β1 induces MORC2 O-GlcNAcylation by enhancing the stability of GFAT. HBP controls the production of UDP-GlcNAc, the donor substrate for OGT, and the first and limiting step of HBP is catalyzed by GFAT [4, 57]. The expression levels of GFAT are elevated in multiple types of human cancer and are associated with a poor patient prognosis [58–63]. Depletion or pharmacological inhibition of GFAT suppresses tumor growth and sensitizes cancer cells to anti-PD1 therapy and cisplatin cytotoxicity [15, 60, 64]. GFAT has been shown to be transcriptionally regulated [5–9], and its enzymatic activities are modulated by phosphorylation [10–12]. In this study, we demonstrated that GFAT is subjected to proteasomal degradation, and TGF-β1 enhances the stability of GFAT through reducing its ubiquitination (Fig. 4). Furthermore, TGF-β1 enhances MORC2 O-GlcNAcylation, which is dependent on GFAT (Fig. 4). However, how TGF-β1 regulates the ubiquitination of GFAT remains to be addressed in the future. In agreement with our results, it has been shown that cytokine interleukin-1 induces O-GlcNAcylation of TAB1 to modulate TGF-β-activated kinase 1 (TAK1)-mediated cytokine release [54]. In addition, previous studies have shown that overexpression of GFAT induces TGF-β1 synthesis in NIH-3T3 fibroblasts [65], and GFAT enzyme activity is necessary for the induction of TGF-β1 expression in mesangial cells [66]. Thus, whether there is a positive feedback loop between GFAT and TGF-β1 to induce MORC2 O-GlcNAcylation in breast cancer cells remains to be addressed in the future.

Third, O-GlcNAcylated MORC2 transcriptionally activates TGF-β1 target genes CTGF and SNAIL. TGF-β signaling contributes to breast cancer progression through, at least in part, transcriptional regulation of tumor metastasis-related genes, such as SNAIL [67] and CTGF [68]. SNAIL is a master regulator of TGF-β-driven epithelial-mesenchymal transition and a strong inducer of breast cancer invasion and metastasis [69–72]. Similarly, CTGF plays a crucial role in the migratory, invasive, metastatic, and angiogenic processes in human breast cancer cells [73, 74]. Moreover, elevated levels of CTGF in primary breast tumors are associated with more advanced features [75]. Through analysis of publicly available RNA-seq databases followed by validation by immunoblotting and qPCR assays, we found that SNAIL and CTGF are transcriptionally regulated by TGF-β1-indcued MORC2 O-GlcNAcylation (Fig. 5). O-GlcNAcylated MORC2 is recruited to SNAIL and CTGF promoter and enhances their activities (Fig. 6 and Supplementary Fig. S7). Furthermore, expression of an O-GlcNAcylation-defective MORC2 or knockdown of CTGF or SNAIL impairs TGF-β1-induced breast cancer cell migration and invasion (Fig. 6 and Supplementary Fig. S8). These results indicate that TGF-β-MORC2 O-GlcNAcylation-CTGF/SNAIL signaling axis is implicated in breast cancer progression.

In conclusion, the findings presented here uncover a previously unrecognized functional and mechanistic role for MORC2 O-GlcNAcylation in breast cancer progression. OSMI-1, an OGT inhibitor, significantly suppresses MORC2-mediated breast cancer cell invasion and metastasis. Therefore, targeting O-GlcNAc signaling may be a potential therapeutic approach for MORC2-mediated breast cancer progression.

## Materials and methods

### Cell cultures and treatment

Human breast cancer cell lines (MCF-7, T47D, and BT549) and human embryonic kidney 293 T (HEK293T) cell line were obtained from the Cell Bank of the Chinese Academy of Sciences (Shanghai, China). MDA-MB-231-derived LM2–4175 cells were kindly provided by Guohong Hu (University of Chinese Academy of Sciences, Shanghai, China). All cell lines were authenticated by detection of mycoplasma, DNA-fingerprinting, isozyme, and cell vitality. Cell lines were expanded and frozen immediately into numerous aliquots after arrival. The cells revived from the frozen stock were used within 10–15 passages and a period of 6 months.

Cells were maintained in DMEM (BasalMedia, #L110) media supplemented with 10% fetal bovine serum (ExCell Bio, #FSP500) and 1×penicillin-streptomycin solution (BasalMedia, #S110B). DMEM media without glucose (Thermo Fisher, #11966025) was used for glucose-starved experiments. For glucose treatment, cells were cultured in glucose- and serum-free medium for 24 h and then incubated with glucose at indicated concentrations for another 24 h. For TGF-β1 treatment, cells were serum-starved for 24 h and then incubated with or without 5 ng/ml recombinant human TGF-β1 (Cell Signaling Technology, #8915LF) for the indicated times. Unless otherwise stated, all reagents were purchased from Sigma-Aldrich. The detailed information for chemical reagents is provided in Supplementary Table S2.

### Expression vectors, plasmid transfection, and lentiviral infection

Myc-DDK-tagged MORC2 was obtained from Origene (#RC200518), and then subcloned into the lentiviral vector pCDH-CMV-MCS-EF1-Puro (System Biosciences, #CD510B-1) or pMSCV-Hyg to generate HA-MORC2 and Flag-MORC2. cDNAs for OGT (#CH806183) and OGA (#CH801095) in pEnter vector were obtained from Vigene Biosciences. cDNA for OGT was subcloned into the lentiviral vector pCDH-CMV-MCS-EF1-Puro (System Biosciences, #CD510B-1) to generate HA-OGT using CloneEZ PCR Cloning Kit (Genscript, #L00339). Site-directed mutagenesis was generated by conventional PCR-based method. Expression vector encoding V5-ubiquitin has been described previously [32]. Generation of MORC2 knockout (KO) cells using single-guide RNA sequences (sgRNA) targeting MORC2 by CRISPR/Cas9 technology has been described previously [27, 30, 32]. The CTGF and SNAI promoter regions (−900 to −2000 and +100 to −500) were synthesized by GENEWIZ Biotech and cloned into a pGL3-basic luciferase reporter vector (Promega, #E1751). All construct sequences were verified by DNA sequencing. The detailed information concerning expression constructs and the primers used for molecular cloning is provided in Supplementary Tables S3 and S4. Transient plasmid transfection was performed using Neofect DNA transfection reagent (TengyiBio, #TF201201) according to the manufacturer’s protocol. Lentiviral infection and generation of stable cell lines were carried out as described previously [27, 30, 32].

### Small interfering RNAs (siRNAs) and transfection

Specific siRNAs targeting OGT, OGA, GFAT, SNAIL, CTGF, and corresponding negative control siRNAs (siNC) were purchased from GenePharma (Shanghai, China), and their targeting sequences are listed in Supplementary Table S5. The siRNA duplexes were transfected into cells using Lipofectamine 2000 transfection reagents (Invitrogen, #11668019) following the manufacturer’s instructions. Knockdown efficiency was determined by immunoblotting analysis after 48 h of transfection.

### Antibodies, immunoblotting, immunoprecipitation assays

All primary and secondary antibodies used in this study are summarized in Supplementary Table S6. Immunoblotting analysis and IP assays were performed as described previously [27, 30, 32]. The optical density of immunoblotting bands was quantified using ImageJ program and was normalized to the corresponding controls.

### Immunofluorescent staining

Immunofluorescent staining was carried out as described previously [27, 30, 32]. Briefly, cells were fixed with 4% methanol-free formaldehyde (Yeasen, #36314ES76) for 20 min and permeabilized with 0.5% Triton X-100 for 20 min at 4 °C. After rinsing with PBS for three times, cells were blocked for 1 h with 5% goat serum and incubated with anti-MORC2 (1:500), or anti-OGT (1:500) antibody in 5% goat serum overnight at 4 °C. Cells were rinsed with PBS three times and incubated with the secondary antibodies conjugated with Alexa 488 or Alexa-568 (1:500) at room temperature for 1 h. Then, cells were washed with PBS for three times, and sealed with a DAPI-containing fluoroshield mounting medium (Abcam, #ab104139). Images were visualized with Leica SP5 confocal microscope and analyzed.

### Purification of recombinant proteins

The GST-tagged constructs in pGEX-6P-1 vector and His-tag constructs in pET-28a vector were transformed into *E. coli* strain BL21 (DE3) and incubated with 0.2 mM IPTG (Invitrogen, #15529019) to induce expression of recombination proteins at 16 °C overnight. GST-tag proteins were purified using Glutathione Sepharose 4B beads (GE Healthcare, #17075601), whereas His-tag proteins were purified using Ni-NTA agarose (TIANGEN Biotech, #WM6-45-655-101), following the manufacturer’s instructions. The purified proteins were immediately used for the experiments or frozen at − 80 °C.

### In vitro O-GlcNAcylation assays

In vitro O-GlcNAcylation assays were performed as described previously [76]. Recombinant Flag-OGT protein purified from HEK293T cells and recombinant His-tagged MORC2 protein purified from *E. coli* were mixed in the reaction buffer (50 mM Tris-HCl pH 7.5, 12.5 mM MgCl_2_, 2 mM UDP-GlcNAc, and 1 mM DTT) in a final volume of 25 μl per sample. The samples were incubated at 37 °C for 24 h. The reaction was resolved with SDS-PAGE, blotted onto a PVDF membrane, followed by immunoblotting with an anti-O-GlcNAc antibody to detect O-GlcNAcylation of MORC2.

### qPCR and ChIP-qPCR

Total RNA was isolated using TRIzol reagent (Invitrogen, #15596018), and 1 μg of RNA was subjected to cDNA synthesis using PrimeScript RT Master Mix (Takara, #RR036). Quantitative real-time PCR (qPCR) was performed using SYBR Premix Ex Taq (Takara, #RR420) following the manufacturer’s instructions. The expression levels of the indicated mRNAs were calculated using the 2^−ΔΔCt^ method and were normalized to internal control GAPDH. Chromatin immunoprecipitation (ChIP) assays were performed using SimpleChIP Enzymatic Chromatin IP Kit (magnetic beads) (Cell Signaling Technology, #9003 S) according to the manufacturer’s instructions. Quantitative results are displayed as corresponding fold change, and anti-rabbit IgG or anti-mouse IgG were used as a negative control. Primers used for qPCR and ChIP assays are listed in Supplementary Tables S7.

### Luciferase assays

Cells were transfected with 200 ng of pGL3, pGL3-SNAIL or pGL3-CTGF expression vectors using Lipofectamine 2000. Renilla luciferase expression vector (pRL) (5 ng) was also transfected into cells as a transfection control. After 48 h of transfection, luciferase assays were performed using a Dual-Luciferase Reporter Assay System (Promega, #E1910) according to the manufacturer’s instructions. The promoter activities were normalized to the corresponding values of Renilla luciferase.

### Transwell migration and invasion assays

Transwell migration and invasion assays were performed as described previously [27] using Boyden chambers with 8 μm pores (Corning Falcon, #353097) and Matrigel Invasion Chambers (Corning BioCoat, #354480), respectively. Medium containing 10% FBS in the lower chamber served as a chemoattractant. The migrated and invaded cells at the bottom of the inserts were fixed with methanol for 30 min and stained with 0.1% crystal violet for 1 h at room temperature. Total number of cells in each chamber was counted. Cells were counted in 10 random fields under microscope.

### Lung metastasis assays

All animal studies were approved by the Institutional Animal Care and Use Committee of Shanghai Cancer Center, Fudan University. For experimental metastasis assays, 2 × 10^6^ MORC2-KO LM2–4175 cells stably expressing pMSCV or Flag-MORC2 (WT or T556A) in 200 μl of PBS were injected in the tail vein of six-week-old BALB/c female nude mice (*n* = 8; Shanghai SLAC Laboratory Animal Co.). All mice were randomly divided into different groups. No statistical methods were used to estimate the number of mice. OSMI-1 (Sigma, #SML1621) was given after one week of injection at a dose of 10 mg/kg/day [77] (dissolved in corn oil with 0.05% DMSO) for four weeks, five days a week and two days off at weekends. Control groups were administrated with the same volume of vehicle control (0.05% DMSO in corn oil). After five weeks of injection, the lungs were excised, fixed in Bouin’s solution overnight, and metastatic lung nodules were counted under a Nikon SMZ1500 stereomicroscope. No blinding was performed during animal experiments.

### Clinical samples and immunohistochemical staining

All procedures were conducted in accordance with the Declaration of Helsinki and International Ethical Guidelines for Biomedical Research Involving Human Subjects and approved by the institutional ethics review board of Fudan University Shanghai Cancer Center. A total of 126 primary breast tumor specimens were obtained from breast cancer patients who underwent surgery at Fudan University Shanghai Cancer Center. These patients did not receive any therapies before surgical operation. Informed consent was obtained from all patients.

IHC staining was performed as described previously [78] using an anti-OGT (Proteintech, #11576, 1:200), anti-O-GlcNAc (ThermoFisher, #MA1–072, 1: 200), anti-MORC2 (Novus, #NBP1–89295, 1:150), anti-SNAIL (Abcam, # ab224731, 1:200) or anti-CTGF (Proteintech, #23936-1-AP, 1:200) antibody. The representative photographs were taken using Olympus BX43 microscope. Interpretation of the IHC results was performed by two independent pathologists who were blinded to the clinicopathological information. Slides were evaluated using light microscopy and a standard semiquantitative immunoreactivity score as described previously [78]. By recording the percentage of positive staining (0, <5%; 1, 5–25%; 2, 26–50%; 3, 50–75%; and 4, >75%) and staining intensity (0, negative; 1, weak; 2, moderate; and 3, strong) for each sample, immunoreactivity score (IRS) (0–12) was calculated by multiplying positive staining percentage with staining intensity. A score of 0–4 was considered as low expression and a score of 5–12 was considered as high expression.

### Statistical analysis

All data are presented as mean ± standard deviation from at least three independent experiments. The unpaired two-tailed Student’s *t* test was used to compare data between two groups using SPSS 20. The probability of survival was estimated with the Kaplan-Meier method and differences between groups were evaluated by the log-rank test. *P* values of less than 0.05 were considered statistically significant.

## Supplementary information

Supplementary information

Reproducibility Checklist

## Data Availability

All data generated or analyzed during this study are included in this article.
