# Supplementary material for: O-GlcNAcylation of MORC2 at threonine 556 by OGT couples TGF-β signaling to breast cancer progression
Source: Cell Death Differ. 2022 Jan 1;29(4):861–73. doi: 10.1038/s41418-021-00901-0 (PMC8991186; doi:10.1038/s41418-021-00901-0)
Supplement: Supplementary file 1 — Supplementary information [file 41418_2021_901_MOESM1_ESM.pdf]

Supplementary Information for

Liu et al. O-GlcNAcylation of MORC2 at threonine 556 by OGT couples TGF-β signaling to breast cancer progression

The Supplementary Information includes

1. Supplementary Figures S1-S9
2. Supplementary Figure legends
3. Supplementary Tables S1-S7

Supplementary Figures and Figure legends

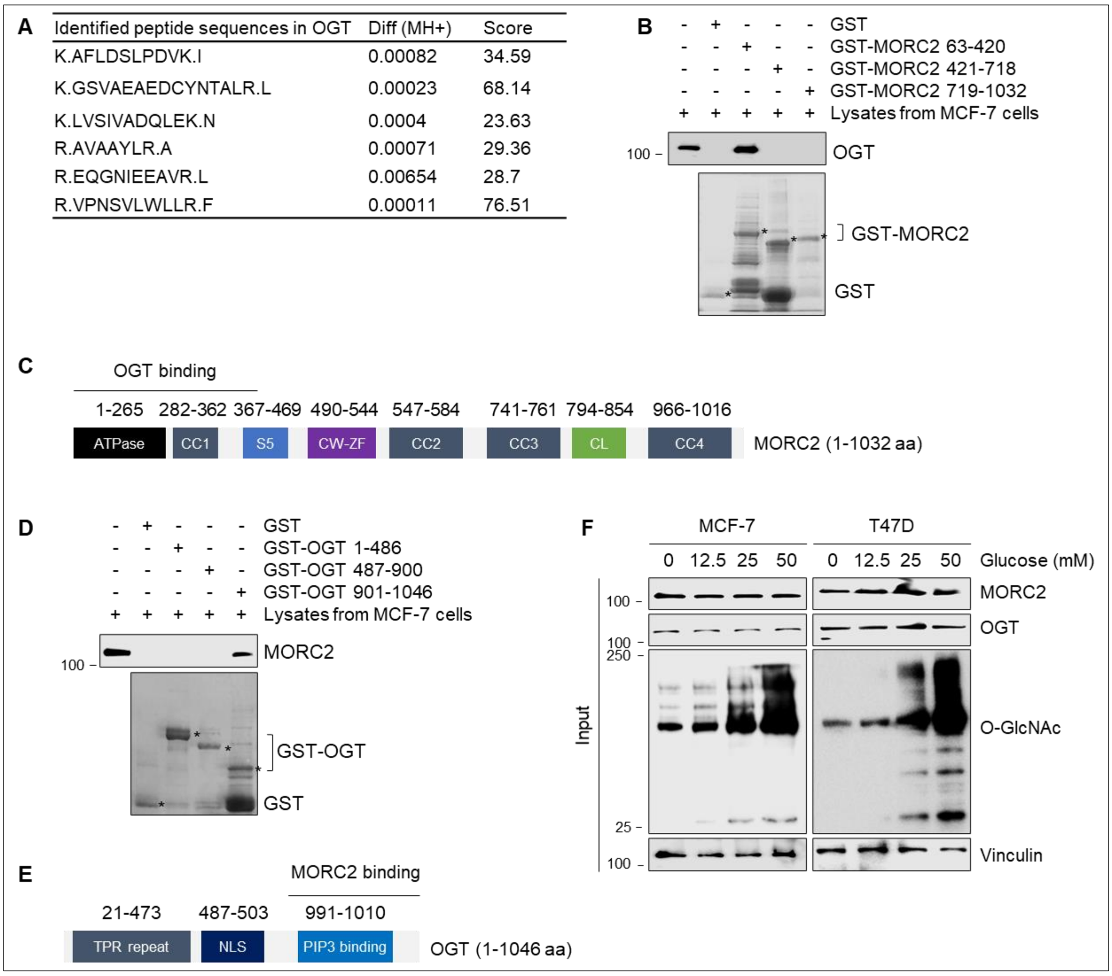

Figure S1. OGT interacts with MORC2

(**A**) Identified peptide sequences in OGT by mass spectrometry. (**B-C**) GST or GST-MORC2 deletion fragments were incubated with lysates from MCF-7 cells. The pull-down protein complex was analyzed by immunoblotting with an anti-OGT antibody. GST or GST-MORC2 proteins were stained by Coomassie blue solution as loading controls (B). Schematic diagram showing the region of MORC2 for OGT binding (C). (**D-E**) GST or GST-OGT deletion fragments were incubated with lysates from MCF-7 cells. The pull-down protein complex was analyzed by immunoblotting with an anti-MORC2 antibody. GST or GST-OGT proteins were stained by Coomassie blue solution as loading controls (D). Schematic diagram showing the region of OGT for MORC2 binding (E). (**F**) MCF-7 and T47D cells were cultured in glucose- and serum-free medium for 24 h, and treated with increasing doses of glucose for 24 h. Immunoblotting analyses were performed with the indicated antibodies.

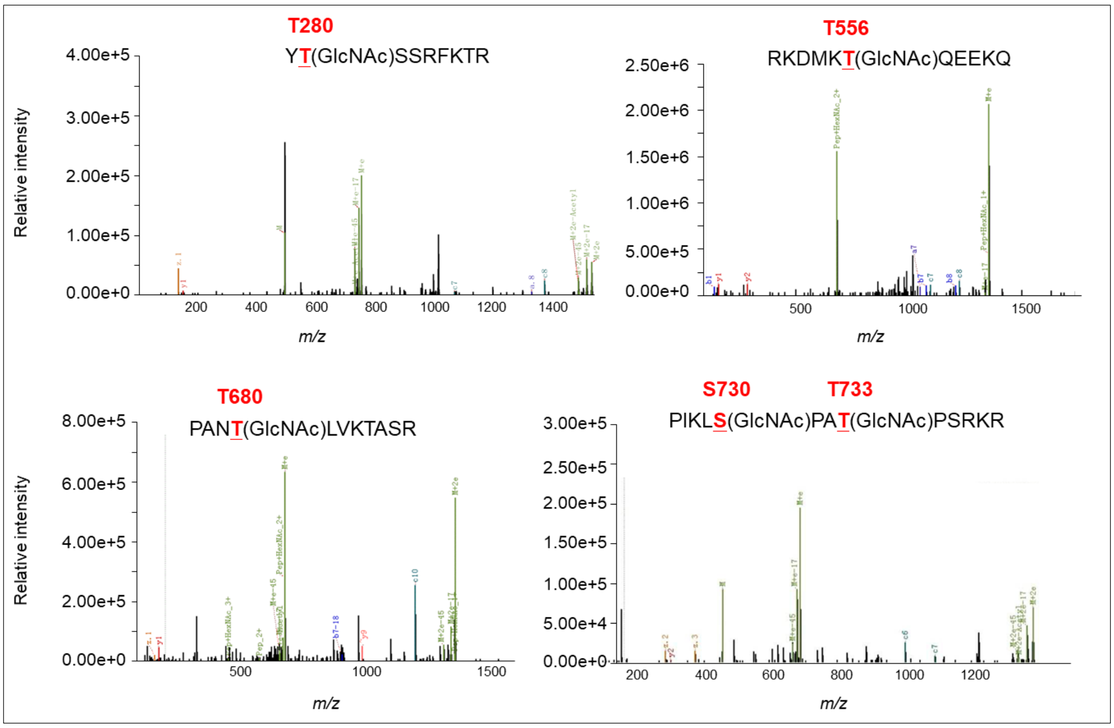

Figure S2. Identification of MORC2 O-GlcNAcylation site(s) by electron transfer dissociation-mass spectrometry (ETD-MS)

HEK293T cells were transiently transfected with Flag-MORC2 and HA-OGT. After 48 h of transfection, After IP using anti-Flag M2 beads and in-gel trypsin digestion, resultant peptides were subjected to electron transfer dissociation-mass spectrometry (ETD-MS) analysis. Identified potential O-GlcNAcylation sites in MORC2 are shown.

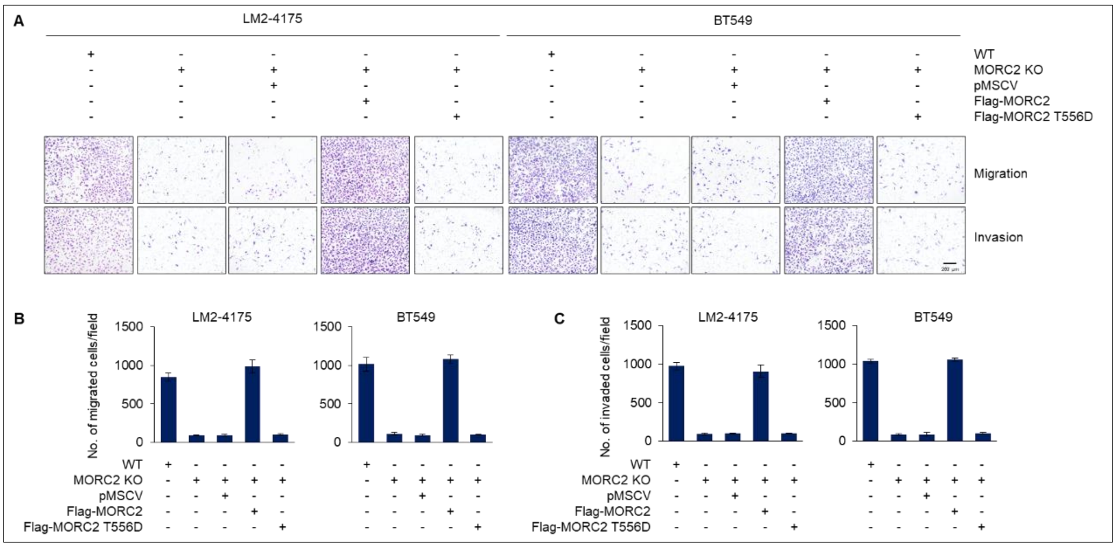

Figure S3. The effects of potential T556 phosphorylation of MORC2 on T556 O-GlcNAcylation-mediated breast cancer cell migration and invasion

T556 residue was replaced with aspartic acid (T556D) to mimic a constitutively phosphorylated MORC2 at T556. Then, endogenous MORC2 in LM2-4175 and BT549 cells were knocked out by CRISPR-Cas9 technology. Empty vector pMSCV or Flag-MORC2 (WT or T556D) were reintroduced into resultant MORC2-KO cells by lentiviral infection. Transwell migration and Matrigel invasion assays were carried out as described as in Materials and methods.

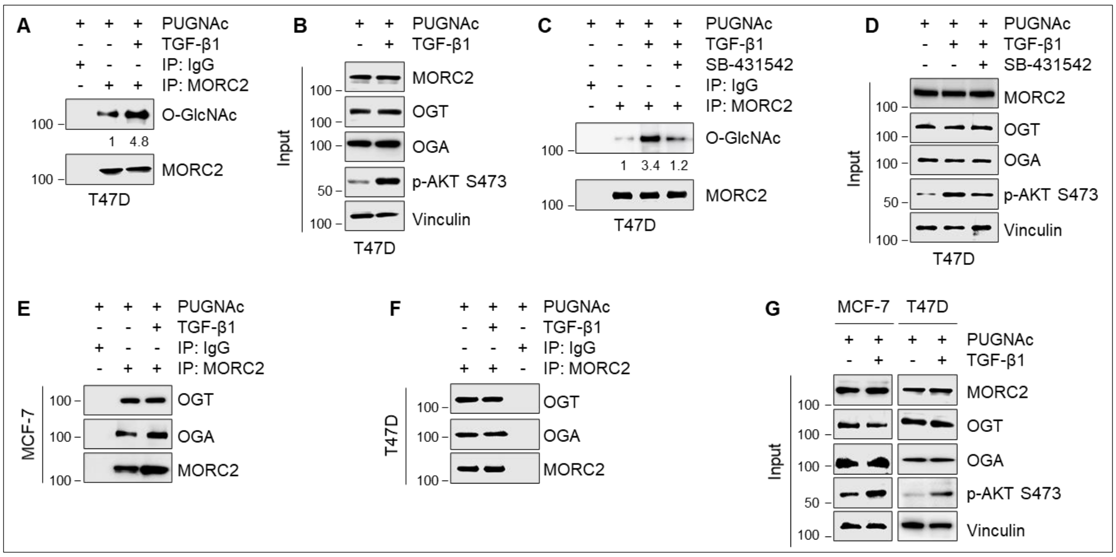

Figure S4. TGF-β1 induces MORC2 O-GlcNAcylation through enhancing the stability of GFAT

(**A-B**) T47D cells were serum-starved for 24 h, treated with or without 5 ng/ml TGF-β1 for 24 h, and then subjected to IP and immunoblotting analysis with the indicated antibodies. In A, O-GlcNAc levels were normalized to the levels of immunoprecipitated MORC2. (**C-D**) T47D cells were serum-starved for 24 h, treated with or without 5 ng/ml TGF-β1 for 24 h alone or in combination with 5 μM TGF-β inhibitor SB431542 for 12 h. Cells were harvested for IP and immunoblotting analysis with the indicated antibodies. In C, O-GlcNAc levels were normalized to levels of immunoprecipitated MORC2. (**E-G**) MCF-7 and T47D cells were serum starved for 24 h, treated with or without 5 ng/ml TGF-β1 for 24 h, and then subjected to IP and immunoblotting analysis with the indicated antibodies.

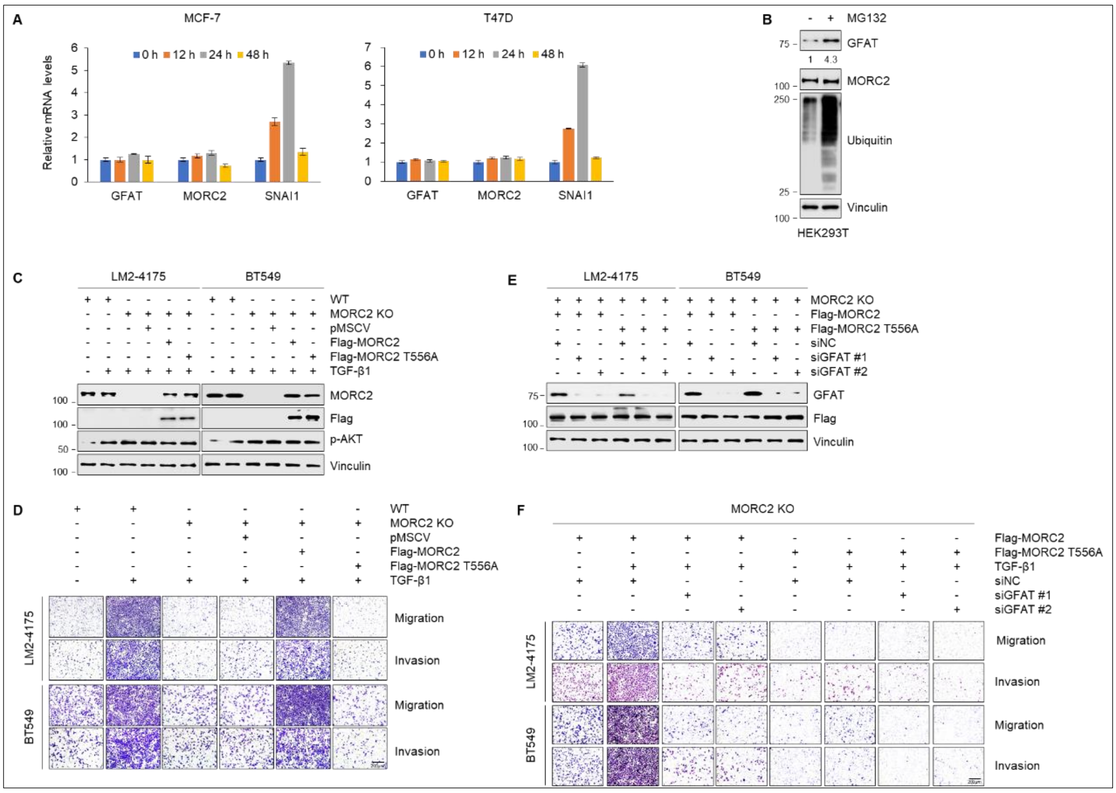

Figure S5. TGF-β1 induces MORC2 O-GlcNAcylation at T556 through enhancing GFAT stability

(**A**) MCF-7 and T47D cells were serum-starved for 24 h, treated with or without 5 ng/ml TGF-β1 for 24 h, and then subjected to qPCR analysis of *GFAT*, *MORC2*, and *SNAIL* mRNA levels. (**B**) HEK293T cells were treated with or without 10 μM MG-132 for 6 h and then subjected to immunoblotting analysis. GFAT levels were normalized to Vinculin levels. (**C-D**) WT and MORC2-KO LM2-4175 and BT549 cells stably expressing pMSCV, Flag-MORC2 or Flag-MORC2 T556A were serum-starved for 24 h, treated with or without 5 ng/ml TGF-β1 for 24 h, and then subjected to Transwell migration and invasion assays. Corresponding quantitative results are shown in Fig. 4O. Expression status of MORC2 in established stable cell lines was verified by immunoblotting analysis (C). Representative images of migrated and invaded cells are shown in D. (**E-F**) MORC2-KO LM2-4175 and BT549 cells stably expressing Flag-MORC2 or Flag-MORC2 T556A were transfected with siNC or two siRNAs targeting GFAT (siGFAT). After 24 h of transfection, cells were serum-starved for 24 h, followed by treatment with or without 5 ng/ml TGF-β1 for 24 h. Transwell migration and invasion assays were performed as described in Materials and Methods. Expression status of Flag-MORC2 and

GFAT in these cell lines was verified by immunoblotting analysis (E). Representative images of migrated and invaded cells are shown in F. Corresponding quantitative results are shown in Fig. 4P.

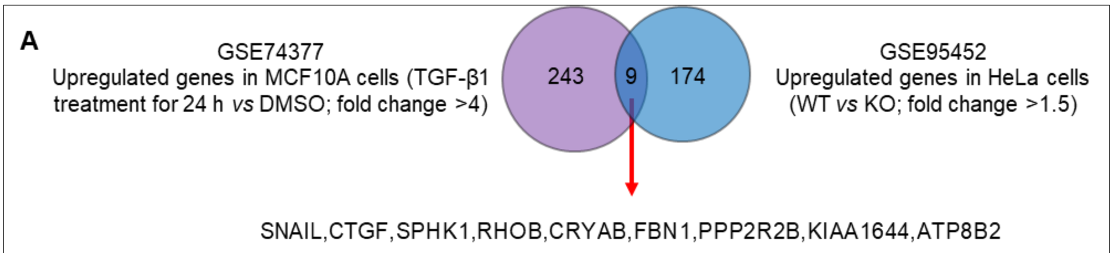

B

Genes	Functions in human cancer (No. of references in PubMed)
SNAIL	5068
CTGF	759
SPHK1	441
RHOB	378
CRYAB	96
FBN1	111
PPP2R2B	24
KIAA1644	1
ATP8B2	2

Figure S6. Analysis of co-regulated genes by MORC2 and TGF-β1

(**A**) Analysis of two publicly available RNA-Seq datasets, including GSE74377 [53] and GSE95452 [19]. According to the preset threshold, 9 commonly upregulated genes by TGF-β1 and MORC2 are indicated. (**B**) The documented functions of those 9 genes in human cancer.

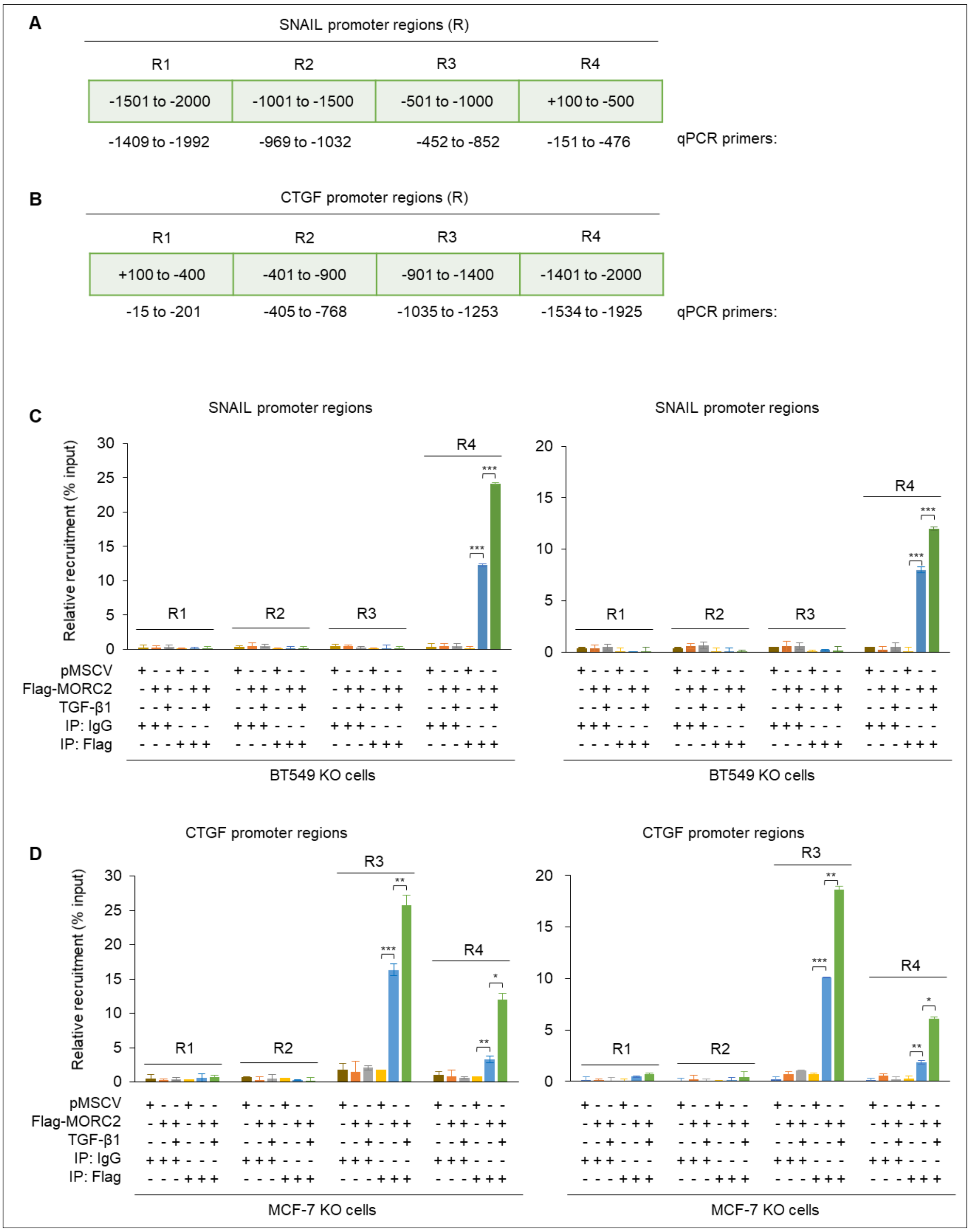

Figure S7. O-GlcNAcylated MORC2 is recruited to the promoter of SNAIL and CTGF

(**A**) The SNAIL promoter (+100 to -2000) was divided into four regions (R) (+100 to -500, -501 to -1000, -1001 to -1500, and -1501 to -2000). qPCR primers were designed against those four regions. (**B**) The CTGF promoter (+100 to -2000) was divided into four regions (R) (+100 to -400, -401 to -900, -901 to -1400, and -1401 to -2000). qPCR primers were designed against those four regions. (**C**) MORC2 KO MCF-7 and BT549 cells stably expressing pMSCV and

Flag-MORC2 were serum-starved for 24 h and then treated with or without 5 ng/ml TGF-β1 for 24 h. ChIP assays were performed with an anti-Flag antibody or IgG, followed by qPCR analysis. Recruitment of Flag-MORC2 to *SNAIL* promoter regions was normalized to the Input. *, $p<0.05$; **, $p<0.001$; ***, $p<0.001$; *NS*, no significance. (**D**) MORC2 KO MCF-7 and BT549 cells stably expressing pMSCV or Flag-MORC2 (WT or T556A) were serum-starved for 24 h, and then treated with or without 5 ng/ml TGF-β1 for 24 h. ChIP assays were performed with an anti-Flag antibody or IgG, followed by qPCR analysis. Recruitment of Flag-MORC2 to the *CTGF* promoter regions was normalized to Input. ***, $p<0.001$; NS, no significance.

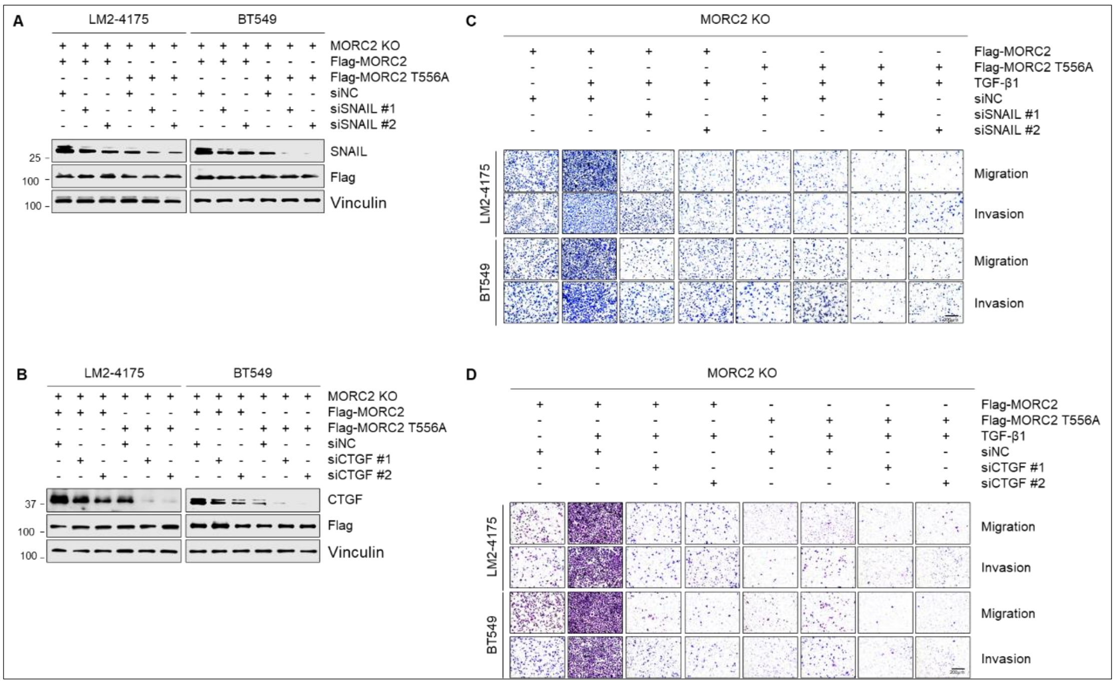

Figure S8. Knockdown of GFAT, SNAIL or CTGF compromises TGF-β1-induced, MORC2 O-GlcNAcylation-mediated breast cancer cell migration and invasion

(**A**) MORC2-KO LM2-4175 and BT549 cells stably re-expressing Flag-MORC2 or Flag-MORC2 T556A were transfected with negative control siRNA (siNC) or two siRNAs targeting SNAIL (siSNAIL). After 24 h of transfection, were serum-starved for 24 h, followed by treatment with or without 5 ng/ml TGF-β1 for 24 h. Immunoblotting analyses were performed with the indicated antibodies. (**B**) MORC2-KO LM2-4175 and BT549 cells stably re-expressing Flag-MORC2 or Flag-MORC2 T556A were transfected with negative control siRNA (siNC) or two siRNAs targeting CTGF (siCTGF). After 24 h of transfection, were serum-starved for 24 h, followed by treatment with or without 5 ng/ml TGF-β1 for 24 h. Immunoblotting analyses were performed with the indicated antibodies. (**C-D**) MORC2 KO LM2-4175 and BT549 cells stably expressing Flag-MORC2 or Flag-MORC2 T556A were transfected with siNC or two siRNAs targeting SNAIL (siSNAIL) (C) or siCTGF (D). After 24 h of transfection, cells were serum-starved for 24 h, followed by treatment with or without 5 ng/ml TGF-β1 for 24 h. Transwell migration and invasion assays were performed as described in Materials and Methods. Corresponding quantitative results are shown in 6I and 6J, respectively.

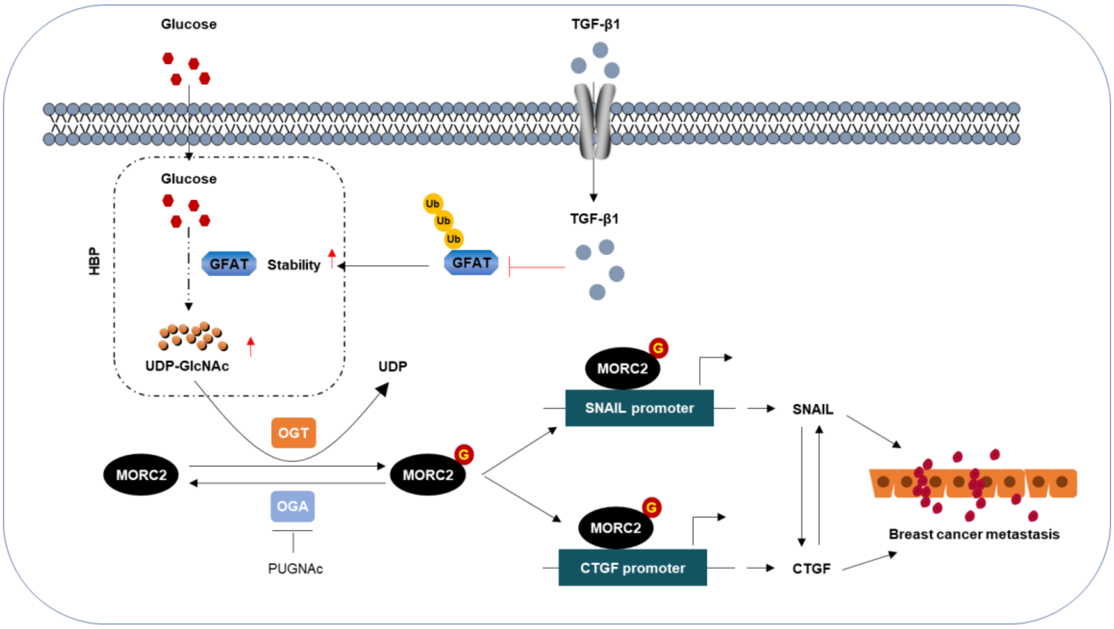

Figure S9. The proposed working model.

MORC2 is O-GlcNAcylated and de-O-GlcNAcylated at the conserved T556 by OGT and OGA, respectively. TGF-β1enhances the stability of GFAT, the rate-limiting enzyme for producing the sugar donor for OGT, to induce MORC2 O-GlcNAcylation. O-GlcNAcylated MORC2 governs the expression of TGF-β1 target genes CTGF and SNAIL, which positively regulate each other, and contributes to breast cancer metastasis.

Supplementary Tables

Table S1. Characterization of clincopathological features of 126 patients with primary breast cancer

Clincopathological factors	No.
Age (yr)	
≤50	64
>50	62
Menopausal status	
Premenopausal	69
Postmenopausal	57
Grade	
I	4
II	52
III	59
Unknown	11
Pathological T-stage	
T1	66
T2	53
T3	4
Tx	3
Pathological N-stage	
N0	81
N1	28
N2	9
N3	8
ER status	
Positive	70
Negative	56
PR status	
Positive	58
Negative	68
HER2 status	
Positive	47
Negative	79

Notes: ER, estrogen receptor; PR, progesterone receptor; HER2, human epidermal growth factor receptor 2

Table S2. Chemical reagents used in this study

Chemical reagents	Vendors	Cat#	Working concentration
OSMI-1	Sigma	SML1621	50 μM
PUGNAc	Sigma	A7229	20 μM
SB431542	Selleck	S1067	5 μM
UDPNAC	Sigma	U4375	2 mM
TGF-β1	CST	8915LF	5 ng/ml
MG-132	Selleck	S2619	10 μM
Insulin	Yeasen	40107ES76	10 μg/ml
EGF	Sigma	E9644	20 ng/ml
Cycloheximide	CST	2112S	100 μg/ml
Hygromycin B	Sigma	V900372	100 μg/ml
Protease inhibitor cocktail	Bimake	B14002	1×
Phosphatase inhibitor cocktail	Bimake	B15001	1×

Table S3. Information for the expression vectors used in this study

Plasmids	Sources	Vectors
Myc-DDK-MORC2	Origene (RC200518)	pCMV6-Entry
HA-MORC2-pCDH	Subcloned	pCDH-CMV-MCS-EF1-Puro
Flag-MORC2-WT	Subcloned	pMSCV-Hyg
Flag-MORC2 T280A	Subcloned	pMSCV-Hyg
Flag-MORC2 T556A	Subcloned	pMSCV-Hyg
Flag-MORC2 T556D	Subcloned	pMSCV-Hyg
Flag-MORC2 T680A	Subcloned	pMSCV-Hyg
Flag-MORC2 S730A	Subcloned	pMSCV-Hyg
Flag-MORC2 S733A	Subcloned	pMSCV-Hyg
GST-MORC2 63-420	Subcloned	pGEX-6P-1
GST-MORC2 421-718	Subcloned	pGEX-6P-1
GST-MORC2 719-1032	Subcloned	pGEX-6P-1
His-MORC2	Subcloned	pET28a (+)
Flag-OGT	Vigenebio (CH806183)	pEnter
HA-OGT	Subcloned	pCDH-CMV-MCS-EF1-Puro
HA-OGT H558A	Subcloned	pCDH-CMV-MCS-EF1-Puro
GST-OGT 1-486	Subcloned	pGEX-6P-1
GST-OGT 487-900	Subcloned	pGEX-6P-1
GST-OGT 901-1046	Subcloned	pGEX-6P-1
pGL3-CTGF-promoter 3&4	GENEWIZ Biotech	pGL3-basic
pGL3-SNAIL-promoter 4	GENEWIZ Biotech	pGL3-basic
Flag-OGA	Vigenebio (CH801095)	pEnter
Lenti-Cas9-blast	Addgene (52962)	pFUGW
LentiGuide-Puro	Addgene (52963)	lentiGuide-Puro
LentiGuide-Puro-MORC2#1	Subcloned	lentiGuide-Puro
LentiGuide-Puro-MORC2#2	Subcloned	lentiGuide-Puro

Table S4. Primers used for molecular cloning of expression vectors

Plasmids	Primers	Sequences
Flag-MORC2 WT	Forward	AATACGGATCCGATGCTTTGCTTTTTGGA TGAT
	Reverse	TATTCCGCGGCCGCTATGGCCAAGACGTC GACGCCTTTTAATCGTCGTCATCCTTGTAA TCGTCCCCCTTGGTGATGAGGTC
Flag-MORC2 T280A	Forward	TACAAGCCCAGGATGTACAAGTACGCCTC AAGCCGTTTC
	Reverse	CGCACGGGTCTTGAAACGGCTTGAGGCGT ACTTGTACAT
Flag-MORC2 T556A	Forward	GGAACATTCAGAAAGGACATGAAGGCCC AGGAAGAGAAG
	Reverse	CAGTTGTTTCTGCTTCTCTTCCTGGGCCTT CATGTCCTT
Flag-MORC2 T556D	Forward	GGAACATTCAGAAAGGACATGAAGGATC AGGAAGAGAAG
	Reverse	CAGTTGTTTCTGCTTCTCTTCCTGATCCTT CATGTCCTT
Flag-MORC2 T680A	Forward	GAGGCACCCCGAAAGCCTGCCAACGCCCT CGTCAAGACT
	Reverse	AGGTCGGGATGCAGTCTTGACGAGGGCGT TGGCAGGCTT
Flag-MORC2 S730A	Forward	AAGACAGAGTCACCCATCAAACTCGCCCC GGCTACCCCT
	Reverse	CCGCTTCCGACTAGGGGTAGCCGGGGCGA GTTTGATGGG
Flag-MORC2 S733A	Forward	TCACCCATCAAACTCTCCCCGGCTGCCCC TAGTCGGAAG
	Reverse	TGCGACACTCCGCTTCCGACTAGGGGCAG CCGGGGAGAG
HA-MORC2	Forward	TTCCTCGAGACTAGTTCT GCCACC ATGGCTTTCACAAATTAC
	Reverse	GGATCCGCGGCCGCTCTTTAAGCGTAGTC TGGGACGTCGTATGGGTAGTCCCCCTTGG TGATGA
GST-MORC2 63-490	Forward	CTGGGATCCCCGGAATTCATGCTTTG CTTTTTGGATGATGGAG
	Reverse	GCGGCCGCTCGAGTCGACTTAGACC AGGTAGGGCACATCAACAAC
GST-MORC2 491-718	Forward	CTGGGATCCCCGGAATTCATGGTCCT GGAGCCTACACACAACAAACA
	Reverse	GCGGCCGCTCGAGTCGACTTATGGA GTCTTGATGACTTTGGGAGA
GST-MORC2 719-1032	Forward	CTGGGATCCCCGGAATTCATGGTGGT GAAGAAGACAGAGTCACCC
	Reverse	GCGGCCGCTCGAGTCGACTTAGTCC CCCTTGGTGATGAGGTC
His-MORC2	Forward	TTGCGGCCGCATGGCTTTCACAAATTACA GC
	Reverse	GGAATTCTCAGTCCCCCTTGGTGATGAG

HA-OGT	Forward	AATAGCGGCCGCATGGCGTCTTCCGTGGGCAAC
	Reverse	TATTTTAGGATCCTGCTGACTCAGTGACTTCAAC
HA-OGT H568A	Forward	GGATATGTGAGTTCCGACTTTGGGAATGCCCCTACTTCTCAC
	Reverse	AATAGACTGCATAAGGTGAGAAGTAGGGGCATTCCCAAAGTC
GST-OGT 1-486	Forward	AATACTCGAGATGGTGCAGAAGGAGAGTCAA
	Reverse	TATTTTAGCGGCCGCCCTACTGCTGTATTGATGAG
GST-OGT 487-900	Forward	AATACTCGAGCAAGTTGCACACAGTGGAGCT
	Reverse	TATTTTAGCGGCCGCTGGCTGAAAAAAGAGATCATT
GST-OGT 901-1046	Forward	AATACTCGAGCCTCCACTGACTCCTACCTCC
	Reverse	TATTTTAGCGGCCGCCAGGCTCCGACCAAGTATAAC

Table S5. Targeting sequences for siRNAs

	Primers	Sequences
siNC	Forward	UUCUCCGAACGUGUCACGUTT
	Reverse	ACGUGACACGUUCGGAGAATT
siOGT#1	Forward	GUUGGCACAUCGAGAAUAUTT
	Reverse	AUAUUCUCGAUGUGCCAACTT
siOGT#2	Forward	CCUGGCUUGUGUAUACUAUTT
	Reverse	AUAGUAUACACAAGCCAGGTT
siOGA#1	Forward	GCAGCAGACAAAGAGGUAUTT
	Reverse	AUACCUCUUUGUCUGCUGCTT
siOGA#2	Forward	GCUCUAAAGCUAGCAUUAATT
	Reverse	UUAAUGCUAGCUUUAGAGCTT
siGFPT#1	Forward	CCUGGAGACCCUAAUCAAATT
	Reverse	UUUGAUUAGGGUCUCCAGGTT
siGFPT#2	Forward	GGAGAGAGUUAUCCAACAATT
	Reverse	UUGUUGGAUAACUCUCUCCTT
siCTGF#1	Forward	GCACCAGCAUGAAGACAUATT
	Reverse	UAUGUCUUCAUGCUGGUGCTT
siCTGF#2	Forward	CCGACUGGAAGACACGUUUTT
	Reverse	AAACGUGUCUUCCAGUCGGTT
siSNAIL#1	Forward	GCCUUCAACUGCAAAUACUTT
	Reverse	AGUAUUUGCAGUUGAAGGCTT
siSNAIL#2	Forward	CCAAUGCUCAUCUGGGACUTT
	Reverse	AGUCCCAGAUGAGCAUUGGTT

Table S6. Antibodies used in this study

Antibodies	Vendors	Cat#	Hosts	Working concentration
OGT	Abcam	ab184198	Mouse	1: 500 (IF)
OGT	Proteintech	11576	Rabbit	1:2000
MORC2	Novus	NBP1-89295	Rabbit	1: 500 (IF)
MORC2	Bethyl	A300-149	Rabbit	1: 1000
OGA	Abcam	ab124807	Rabbit	1: 1500
O-GlcNAc	ThermoFisher	MA1-072	Mouse	1: 1000 (WB)<br>1:200 (IF)
His	CST	12698	Rabbit	1: 1500
HA	CST	3724	Rabbit	1: 1500
Flag	Sigma	F1804	Mouse	1: 3000
Mouse IgG, HRP-linked	CST	7076	Mouse	1: 1000
Rabbit IgG, HRP-linked	CST	7074	Rabbit	1: 1000
Mouse IgG (Alexa Fluor 555 Conjugate)	CST	4409	Mouse	1: 500 (IF)
Rabbit IgG (Alexa Fluor 488 Conjugate)	CST	4412	Rabbit	1: 500 (IF)
Mouse IgG (Alexa Fluor 488 Conjugate)	CST	4408	Mouse	1: 500 (IF)
Rabbit IgG (Alexa Fluor 555 Conjugate)	CST	4413	Rabbit	1: 500 (IF)
GST	GNI	GNI4110-GT	Mouse	1: 1000
CTGF	Proteintech	23936-1-AP	Rabbit	1: 500
GFAT	Abcam	ab125069	Rabbit	1: 500
SNAIL	CST	3879S	Rabbit	1: 1000
Vinculin	Sigma	V9131	Mouse	1: 3000
p-AKT S473	CST	4060T	Rabbit	1: 1000
Myc	CST	2276S	Mouse	1: 3000
MPP8	Proteintech	16796-1-AP	Rabbit	1: 1000
SETDB1	Proteintech	11231-1-AP	Rabbit	1: 1000
PPHLIN	Abclonal	A16295	Rabbit	1: 1000
TASOR	Abclonal	A13187	Rabbit	1: 1000

Table S7. Primers for qPCR analyses

	Primers	Sequences
GFPT	Forward	GGAATAGCTCATACCCGTTGG
	Reverse	TCGAAGTCATAGCCTTTGCTTT
MORC2	Forward	CCTATGCCGCTGTGCTCTAT
	Reverse	TGCTTTCTTCACCTCCTGCT
SNAIL	Forward	TCGGAAGCCTAACTACAGCGA
	Reverse	AGATGAGCATTGGCAGCGAG
CTGF	Forward	ACCGACTGGAAGACACGTTTG
	Reverse	CCAGGTCAGCTTCGCAAGG
GAPDH	Forward	CTGGGCTACACTGAGCACC
	Reverse	AAGTGGTCGTTGAGGGCAATG
CTGF-promoter R1 (ChIP)	Forward	AAAAAGTTGAGGGCCAAGTTGC
	Reverse	GAATGGCCAGCAAAGGGGTG
CTGF-promoter R2 (ChIP)	Forward	AGCCGATCTTTGCACCACA
	Reverse	GGAGTGAGGTCAGGACAAGG
CTGF-promoter R3 (ChIP)	Forward	GAACCCCCTTTGCATCCCAG
	Reverse	GTGACTCAGGATGCAGTCTCC
CTGF-promoter R4 (ChIP)	Forward	TGGACAGAACAGGGCAAACTT
	Reverse	CCTCATCAACTCACACCGGA
CTGF-promoter R3&R4 (ChIP)	Forward	AGGTGAACCCCCTTTGCATC
	Reverse	CTCAGCGGGGAAGAGTTGTT
SNAIL-promoter R1 (ChIP)	Forward	ATGAAAGGAAGCCAGCGTGA
	Reverse	CCGTCCCGTGTCAATTTAGC
SNAIL-promoter R2 (ChIP)	Forward	AGGACGATTTTGTTCACGGC
	Reverse	GAGGAGGGACCTGGTTAGAGT
SNAIL-promoter R3 (ChIP)	Forward	GGAAGCTGCTCTCTAGGAGTT
	Reverse	ATTATCAAGGGAAAAGGCCCGA
SNAIL-promoter R4 (ChIP)	Forward	TGCGTTTCCCTCGTCAATGC
	Reverse	GAGGAAAGAGCGCGGCATA
